# Metabolically diverse microorganisms mediate methylmercury formation under nitrate-reducing conditions in a dynamic hydroelectric reservoir

**DOI:** 10.1038/s41396-023-01482-1

**Published:** 2023-07-26

**Authors:** Benjamin D. Peterson, Brett A. Poulin, David P. Krabbenhoft, Michael T. Tate, Austin K. Baldwin, Jesse Naymik, Nick Gastelecutto, Katherine D. McMahon

**Affiliations:** 1 Department of Civil and Environmental Engineering, University of Wisconsin - Madison, Madison, WI 53706 USA; 2 Department of Bacteriology, University of Wisconsin - Madison, Madison, WI 53706 USA; 3 Department of Environmental Toxicology, University of California - Davis, Davis, CA 95616 USA; 4 U.S. Geological Survey, Upper Midwest Water Science Center, Mercury Research Laboratory, Madison, WI 53726 USA; 5 U.S. Geological Survey, Idaho Water Science Center, Boise, ID 83702 USA; 6 Idaho Power Company, Boise, ID 83702 USA

**Keywords:** Biogeochemistry, Water microbiology

## Abstract

Brownlee Reservoir is a mercury (Hg)-impaired hydroelectric reservoir that exhibits dynamic hydrological and geochemical conditions and is located within the Hells Canyon Complex in Idaho, USA. Methylmercury (MeHg) contamination in fish is a concern in the reservoir. While MeHg production has historically been attributed to sulfate-reducing bacteria and methanogenic archaea, microorganisms carrying the *hgcA* gene are taxonomically and metabolically diverse and the major biogeochemical cycles driving mercury (Hg) methylation are not well understood. In this study, Hg speciation and redox-active compounds were measured throughout Brownlee Reservoir across the stratified period in four consecutive years (2016–2019) to identify the location where and redox conditions under which MeHg is produced. Metagenomic sequencing was performed on a subset of samples to characterize the microbial community with *hgcA* and identify possible links between biogeochemical cycles and MeHg production. Biogeochemical profiles suggested in situ water column Hg methylation was the major source of MeHg. These profiles, combined with genome-resolved metagenomics focused on *hgcA*-carrying microbes, indicated that MeHg production occurs in this system under nitrate- or manganese-reducing conditions, which were previously thought to preclude Hg-methylation. Using this multidisciplinary approach, we identified the cascading effects of interannual variability in hydrology on the redox status, microbial metabolic strategies, abundance and metabolic diversity of Hg methylators, and ultimately MeHg concentrations throughout the reservoir. This work expands the known conditions conducive to producing MeHg and suggests that the Hg-methylation mitigation efforts by nitrate or manganese amendment may be unsuccessful in some locations.

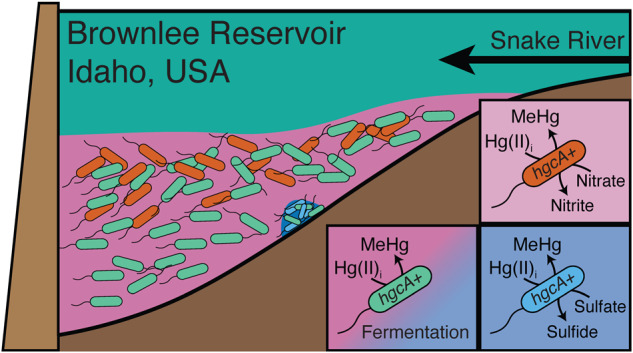

## Introduction

Reservoirs provide critical drinking water supply, flood control, and renewable energy. However, they also have significant impacts on biogeochemical cycles, in part due to thermal and redox stratification facilitated by the riverine-to-lacustrine transition and abundance of terminal electron acceptors (TEAs) and labile organic carbon [1]. This makes reservoirs known hotspots for microbial methylation of inorganic mercury (Hg(II)_i_) to toxic methylmercury (MeHg) [2, 3]. MeHg can be incorporated into the food web [4, 5], partition to particles [6], or be exported downstream as the hypolimnion erodes and the reservoir mixes [7, 8], leading to Hg accumulation in downstream food webs [9]. Understanding where MeHg is produced in reservoirs and identifying the primary biogeochemical drivers of MeHg production is critical to predict Hg impacts on aquatic food webs and manage Hg-impaired reservoirs. Both sulfide and dissolved organic matter (DOM) composition and concentration control Hg(II)_i_ geochemistry and bioavailability for methylation [10, 11]; however, the influence of major biogeochemical cycles on the ability of the resident microbial community to produce MeHg (termed “Hg methylation capacity”) is a key knowledge gap [12]. MeHg production has been historically associated with sulfate-reducing bacteria (SRB) [3, 13], iron-reducing bacteria [14], and/or methanogens [15, 16]. This has led to MeHg mitigation strategies aimed at increasing the redox status of impacted systems, either via oxygenation [17] or addition of other TEAs higher on the redox ladder such as nitrate [18, 19] or manganese (Mn) [20, 21]. MeHg production is linked to biogeochemical cycles by microbes carrying the *hgcAB* gene cluster [22]; the discovery of this genetic marker has greatly expanded our understanding of the diversity of *hgcA*-carrying microbes [16, 23–27], including some aerobic or microaerobic organisms [25, 28, 29]. MeHg production has been observed in oxic environments [30–33] and is independent of methanogenesis and sulfate reduction under anoxic conditions at some sites [14, 34]. Presently, the relevance of putative “high-redox” Hg-methylating organisms to environmental MeHg production is not well understood.

In this study, we evaluated the underlying microbial and geochemical processes modulating MeHg formation in Brownlee Reservoir, a Hg-impaired hydroelectric reservoir within the Hells Canyon Complex (HCC) on the Snake River in Idaho, USA [35]. Over four years with contrasting hydrologic conditions, samples were collected with high spatial and temporal coverage to determine redox conditions and Hg concentration and speciation under various stages of thermal and redox stratification. Genome-resolved metagenomic sequencing was used to identify the taxonomy and metabolic capacity of *hgcA*-carrying (hgcA+) microbes. Overall, this study provides insight into the location and extent of MeHg production and identifies possible biogeochemical drivers of MeHg production in a reservoir system with dynamic hydrology and biogeochemistry. The data provide key information on MeHg formation that aids a larger project to develop comprehensive conceptual and quantitative models of Hg cycling in the river-reservoir system to aid management decisions [6, 7, 35–37].

## Materials and methods

### Site description and sampling

Brownlee Reservoir is the most upstream reservoir within the HCC, spans from river mile (RM) 345 (inflow) to RM284 (outflow through Brownlee Dam) [7, 38], and has a maximum depth of 91 m. The “transition zone” between the riverine and lacustrine zones stretches from the Brownlee Reservoir inflow to approximately RM308, depending on Snake River streamflow and reservoir water surface elevation [39, 40]. Biogeochemical profiles were collected at least monthly from the water column over the stratified period from 2016 to 2019 using trace-metal clean methods with either a Teflon sampling line and a peristaltic pump or an acid-washed Go-Flo sampler (General Oceanics, Miami, FL). Multiparameter sonde profiles were collected concurrent to water sampling. Samples for filter-passing Hg species and metals were collected up to one meter above the sediment:water interface (SWI) as follows. First, a shallow (~30 cm) sediment core was collected using an HTH sediment corer with a 1.5 m core barrel; then, acid-washed Teflon tubing was inserted into the overlying water and water samples were removed in 2–3 intervals, representing distinct layers of water above the SWI. Porewater samples were collected from the top 5 cm of sediment cores [41]. Sample collection protocols, analytical methods, and all physical and geochemical data are available in U.S. Geological Survey data releases [37, 41] and summarized in the Supporting Information and Table S1.

### DNA extraction and sequencing

DNA samples for metagenomic sequencing were collected using 0.22 µm Sterivex filters in 2017, 2018, and 2019. Filters were removed from the cartridges and cells were lysed using physical and chemical lysis methods; then, DNA was extracted using phenol:chloroform and purified using isopropanol precipitation [26, 42]. Library preparation was performed in the Functional Genomics Laboratory and sequencing performed in the Vincent J. Coates Genomics Sequencing Laboratory (QB3, Berkeley, CA). Inserts approximately 600 bp in length were used to generate sequencing libraries with a Kapa Biosystem Library Prep kit (Roche Sequencing and Life Science, Kapa Biosystems, Wilmington, MA). 150 bp paired-end reads were generated on a NovaSeq or HiSeq 4000 (Illumina, San Diego, CA).

### Metagenome assembly, binning, and annotation

Metagenomic metadata and read counts are in Table S2. Reads were trimmed and merged using fastp (v0.20.1) [43]. Metagenomes were clustered by kmer content and coassembled with metaSPADes (v3.14.1) [44]. Assembly statistics are shown in Table S3. Open reading frames (ORFs) were predicted using Prodigal (v2.6.3) [45] and bowtie2 (v2.6.3) was used for read mapping [46]. Gene and bin abundances were calculated as the average read coverage over each nucleotide, then normalized to the median read coverage of 16 ribosomal protein (rp16) genes in each metagenome [47]. Bins were generated with CONCOCT [48] and those containing an *hgcA* gene were manually curated in anvi’o (v6.2) [49]. Bins were grouped into metagenome operational taxonomic units (mOTUs) that shared 98% average nucleotide identity and 50% alignment. The mOTU taxonomies were estimated using GTDB-TK [50]. Metabolic genes were identified using Hidden Markov Models (HMMs) and confirmed phylogenetically (Table S4). Metabolic annotations of mOTUs were done using kofamscan [51], a custom HMM set with hmmer [52], METABOLIC [53], and FEET [54]. Major terminal electron-accepting process (TEAP) gene annotations were confirmed phylogenetically. Phylogenetic trees of mOTUs were based on rp16 gene alignments [47] and built with RAxML [55]. Raw metagenomes are available through NCBI (BioProject: PRJNA878929). HgcA sequences, *hgcA* sequences, and hgcA+ bins are available online at FigShare under project #158018.

### *hgcA* identification and classification

HgcA amino acid sequences were identified in the assembly ORFs using a custom HMM [26], manually screened for the cap helix and transmembrane domains [22], and dereplicated using CD-HIT with a 97% identity cutoff [56]. HgcA sequences were aligned to references from Hg-MATE (v1.01142021) [57] with MUSCLE (v3.8.31) [58]. A maximum-likelihood tree was generated from this alignment using RAxML (v8.2.11) [55] and mid-point rooted with phangorn [59]. HgcA sequences were also classified using an established workflow [57, 60]. Using the autoclassification, HgcA phylogeny, and hgcA+ mOTU phylogeny, each *hgcA* gene was assigned a taxonomic classification. When possible, one of four predicted metabolic guilds was assigned to each *hgcA* gene: high-redox respiratory organism (HRRO), SRB, methanogen (MET) or fermentative (FERM). For details of metabolic assignment, see Table S5. Linear regression of MeHg to *hgcA* abundance was performed on log-log transformed data using the “lm” function in R (v4.1.3).

## Results and discussion

### Hydrologic and biogeochemical conditions

**Fig. 1:**
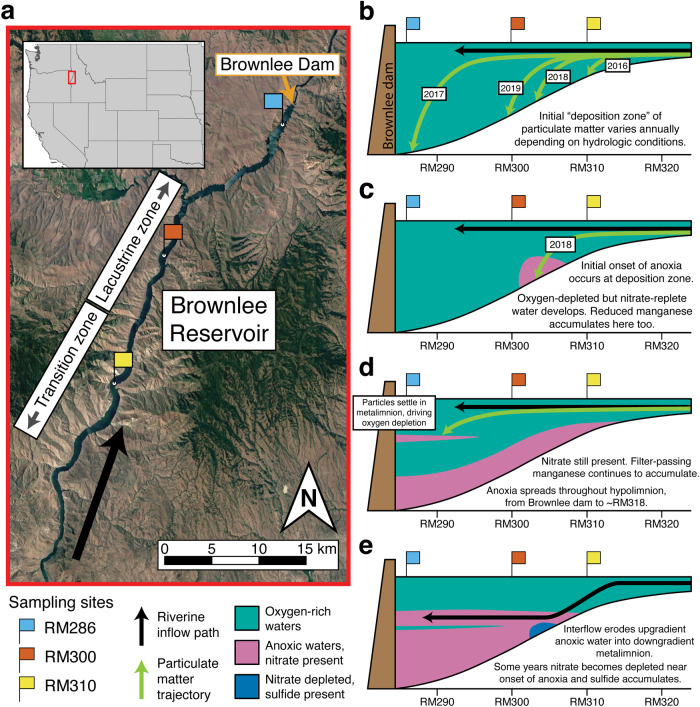
Spatial and temporal changes in redox conditions within Brownlee Reservoir show interannual variation. Map of sampling locations in Brownlee Reservoir and the surrounding watershed (**a**) and schematic of seasonal and interannual variation in anoxia progression (**b**–**e**). Samples for this study were collected from three sites along a 24-mile stretch in Brownlee Reservoir (**a**). Inset shows the location of the reservoir (red box) in the western United States. Particulate organic matter from the Snake River, primarily consisting of autochthonous cyanobacterial and algal biomass, is deposited to different areas of Brownlee Reservoir, in the “deposition zone”, depending on hydrologic conditions (**b**). Panels (**c**) through (**e**) depict redox progression based on 2018 conditions. Anoxia in Brownlee Reservoir initially develops at the deposition zone, where nitrate is still abundant and filter-passing manganese (Mn) starts to accumulate (**c**). Anoxia spreads throughout the hypolimnion and upgradient into the transition zone, until it stretches over 30 river miles (**d**). Nitrate is present throughout much of the hypolimnion during this time. Additionally, particulate organic matter settling at the strongest density gradients in the metalimnion near the dam fuel high microbial activity, resulting in a mid-water column parcel of oxygen-depleted water. Finally, during late stratification, inflowing water begins to cool and sink below the epilimnion of the reservoir, resulting in interflow that erodes upgradient oxygen-depleted waters into the downgradient metalimnion and eventually through the dam (**e**). In some years, nitrate becomes depleted near the initial onset of anoxia and sulfide begins to accumulate. In the winter, the inflowing water cools further, resulting in underflow that fully erodes the hypolimnion, resulting in a fully mixed and oxic lacustrine zone.

The hydrologic and biogeochemical conditions were evaluated at three sites spanning the lacustrine (RM286, RM300) and transition zone (RM310) of Brownlee Reservoir (Fig. 1a) [39, 40]. The lacustrine sites are divided by reservoir strata into the epilimnion, metalimnion, and hypolimnion based on temperature differences as done previously [39]. Parallel studies in Brownlee Reservoir documented processes influencing seasonal anoxia (defined here as oxygen <0.5 mg/L) [39], MeHg accumulation, Hg aqueous-particulate partitioning [6] and MeHg export [7, 36], which inform interpretations in this study. Brownlee Reservoir was thermally mixed and fully oxygenated during winter (Fig. 1b) prior to seasonal stratification from March/April to November/December. During spring, particulate material in the Snake River was mobilized by high flow conditions and transported downgradient towards Brownlee Reservoir. As flow velocities decreased in the transition zone of Brownlee Reservoir, the particulate material was deposited to the benthos [35, 61]. During the study period (2016–2019), interannual variability in the spring flow conditions of the Snake River entering Brownlee Reservoir (Fig. S1) influenced the location of the “deposition zone” of particulate material (Fig. 1b). Particulate deposition is interpreted to have driven heterotrophic microbial activity, which led to hypolimnetic anoxia (Fig. S2). Due to the variation in the location of the deposition zone, the initial site of anoxia varied year-to-year (Figs. S2, 1c). Over the summer stratified period, anoxia spread longitudinally up- and downgradient in Brownlee Reservoir (covering 30 river miles) and vertically in the water column (>40 m above the SWI; Fig. 1d). During fall, cooler inflowing water dove below warmer epilimnetic water; these interflow events eroded anoxic waters from the transition zone and metalimnion, resulting in sequential destratification (Fig. 1e) [6, 36]. During late fall and early winter, further cooling of the Snake River resulted in underflow and complete destratification and oxygenation of the hypolimnion of the reservoir [39].

Each year of the study, MeHg accumulated in the hypolimnion, metalimnion, and bottom waters of the transition zone (Fig. S3). MeHg concentrations in the epilimnion were rarely above 0.05 ng/L, despite previous evidence for MeHg production in oxic waters in Brownlee Reservoir [30]. There was notable interannual variation in the location of initial accumulation and maximum concentration of MeHg, which followed the initial onset of anoxia (Figs. S2, S3). However, in all sampling years, filter-passing MeHg accumulated in the hypolimnion at all sampled locations over the stratified period (Fig. S3). There was also consistent MeHg accumulation in the metalimnion, albeit to lower concentrations (Fig. S3). MeHg accumulation in the reservoir hypolimnion is interpreted to be heavily influenced by in-reservoir production for two reasons. First, MeHg concentrations entering Brownlee Reservoir and in the epilimnion during summer and fall were notably lower (Figs. S3, S4) than the reservoir hypolimnion. Second, particulate MeHg loading, which occurs in the spring before hypolimnetic MeHg accumulation [7], is expected to have little effect on filter-passing MeHg [62]. Conversely, unfiltered Hg(II)_i_ at the inflow was relatively high, but filter-passing and particulate Hg(II)_i_ within Brownlee Reservoir showed little change with depth (Figs. S5, S6), suggesting rapid Hg(II)_i_ removal in the lacustrine zone, either through partitioning to particles and subsequent settling or conversion to MeHg [6]. Demethylation rates under dark conditions in Brownlee Reservoir are slow, further supporting methylation as the dominant control on MeHg concentrations [30]. Overall, we assert that in-reservoir production plays an important role in MeHg accumulation in the metalimnion and hypolimnion.

**Fig. 2:**
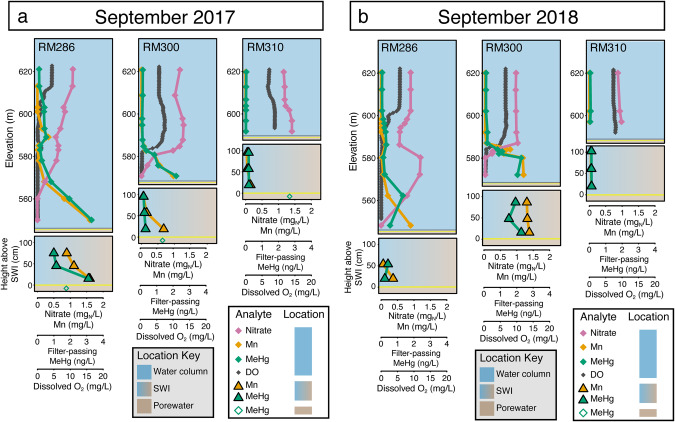
Redox profiles from Brownlee Reservoir during late stratification show evidence for water column methylmercury (MeHg) production under nitrate-reducing conditions. Profiles shown correspond to fall intensive trips from September 2017 (**a**) and September 2018 (**b**) when samples for metagenomic sequencing were collected. Complete water chemistry data are available in the associated U.S. Geological Survey data release. Upper panels show water chemistry parameters in the water column. In the lower panels, the filled triangles represent water chemistry data from the water immediately overlying the sediment:water interface (SWI). The open diamonds show porewater concentrations, only available in 2017. All manganese (Mn) and MeHg values are for the filter-passing fraction. The yellow line shows the elevation of the sediment:water interface (SWI) and the black line on the upper panels denotes the top of the SWI plot. The shading represents the areas designated as the water column, water overlying the SWI, or porewater.

In-reservoir MeHg production could either occur in the water column, where the MeHg is observed, or in the sediments, which would require that the MeHg subsequently diffuses across the SWI into the hypolimnion. Sediment diffusion was evaluated as a potential in-reservoir source of MeHg by measuring MeHg depth profiles of the water immediately overlying (<1 m) the SWI and into the sediment porewater. Porewater MeHg levels were relatively high (1–4 ng/L), indicating that benthic MeHg production is likely. However, MeHg concentrations just above the SWI were comparable to or lower than hypolimnetic MeHg concentrations (Fig. 2, S7). If diffusion of MeHg across the SWI was responsible for elevated MeHg 40 m above the SWI, MeHg concentrations in the porewater and the water overlying the SWI should be far higher than the hypolimnetic water. While sediment diffusion is often cited as a source for hypolimnetic MeHg accumulation [63–65], studies that have investigated MeHg sources in lacustrine systems with an anoxic hypolimnion by looking in situ at water overlying the SWI all indicate that diffusion of sediment-derived MeHg is not a prominent source of MeHg to the hypolimnion [66–69]. Additionally, advective flux across the SWI has been shown in other systems to overwhelm diffusive flux of MeHg [70]; given the considerable hydraulic head in the lacustrine section of Brownlee Reservoir, it is likely that there is advective flux of water out of the bottom of the reservoir in the deep lacustrine parts of the reservoir, which could preclude sediment-derived MeHg from diffusing across the SWI. Taken together, our observations are inconsistent with a diffusive gradient of MeHg across the SWI and suggest a limited influence of benthic MeHg production on water column MeHg accumulation. Rather, we propose that in-reservoir water column Hg methylation is the dominant source of filter-passing MeHg, consistent with other freshwater lacustrine systems [26, 27, 66, 67, 71, 72].

MeHg accumulated throughout the reservoir under relatively high redox conditions (Figs. 2, S7). Biogeochemical TEAs are described below step-wise from high- to low-redox couple. In each year of the study, hypolimnetic nitrate concentrations progressively decreased over the reservoir stratification period (Fig. S8) concurrent with oxygen consumption (Fig. S2) and MeHg accumulation (Fig. S3). Of the 161 anoxic samples, only 15 exhibited nitrate <0.05 mg_N_/L. Samples where dissolved oxygen was <0.5 mg/L and nitrate >0.05 mg_N_/L are henceforth referred to as “oxygen-depleted”, while samples where nitrate <0.05 mg_N_/L will be referred to as “nitrate-depleted”. In 2016, nitrate depletion was not observed; in all other study years, nitrate depletion was only observed at the bottom of the hypolimnion in late summer, well after MeHg had started to accumulate (Figs. S8, S3). Each year, filter-passing Mn accumulated in the hypolimnion over the reservoir stratification period (maximum = 1.6 mg/L; Fig. S9). However, particulate Mn in the water column was low, rarely above 0.2 mg/L (Fig. S10), which suggests that filter-passing Mn originated from rapid reductive dissolution of Mn(III/IV)-oxides on settling particles [68]. There was no evidence of Fe(III) reduction to Fe(II) in the water column, as filter-passing Fe below the epilimnion was once up to 0.061 mg/L, but otherwise remained under 0.025 mg/L (Figs. S11, S12). Sulfate concentrations were relatively high in the reservoir (~20–60 mg/L), but depth profiles showed no sign of significant sulfate consumption (Fig. S13). Inorganic sulfide, the product of dissimilatory sulfate reduction, was only detected in four samples across the four years of this study, twice at RM286 in 2017 and once each at RM300 and RM310 in 2018 (Table S1). Sulfide was not detected in any samples where nitrate >0.05 mg_N_/L. Further, thiosulfate, produced by incomplete dissimilatory sulfate reduction or inorganic sulfide oxidation [73], was at or less than the daily detection limit (0.008–0.05 mg/L) in all 135 samples where it was measured except for two samples where sulfide was present and/or nitrate was completely depleted (Table S1). Taken together, the geochemical data suggest that denitrification is the dominant TEAP in the oxygen-depleted regions of Brownlee Reservoir during stratification and MeHg accumulation.

**Fig. 3:**
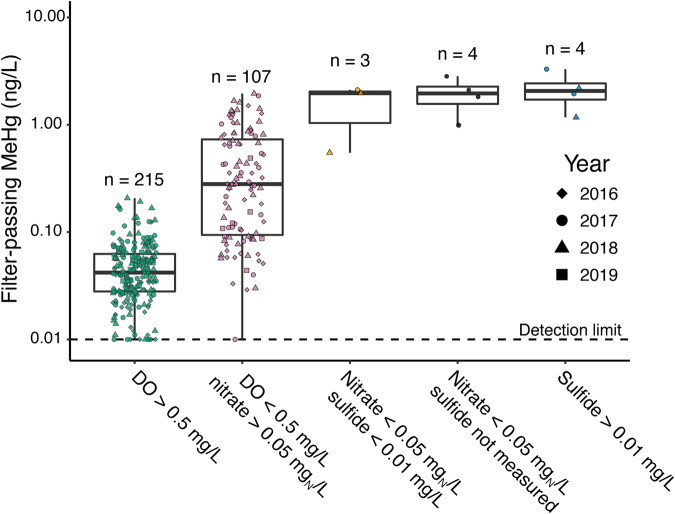
Methylmercury (MeHg) concentrations by redox status of the water column. Redox status was reported as a qualitative category based on measured dissolved oxygen (DO), nitrate, and sulfide concentrations. Boxplots represent median and interquartile range (IQR). Boxplot whiskers extend to the lowest and highest values within 1.5 times the IQR. Shapes represent the year the sample was collected. Colors of the points are described on the x-axis of (**a**) and correspond to the redox status calculated previously (Table S1). Detection limit for MeHg was 0.01 ng/L.

Each sample location was assigned a redox status based on the water chemistry of the redox-active constituents (Table S1); MeHg concentrations were then grouped by redox status (Fig. 3). MeHg was consistently low when dissolved oxygen was >0.5 mg/L (maximum = 0.21 ng/L; mean = 0.05 ± 0.03; *n* = 215). Although MeHg concentrations were highest under nitrate depletion/sulfide accumulation conditions (*n* = 11 in total), samples with detectable nitrate still showed appreciable MeHg accumulation (maximum = 1.95 ng/L; mean = 0.47 ± 0.47 ng/L; *n* = 107). We then investigated potential factors controlling MeHg production in Brownlee Reservoir. MeHg did not correlate with either DOC concentration (*p* = 0.156; Fig. S14a) or DOM SUVA_254_ (*p* = 0.264; Fig. S14b), and sulfide was not detected in most samples. As DOM character and concentration and sulfide concentration are the primary determinants of Hg(II)_i_ bioavailability [10, 11, 74, 75], we interpret these observations to indicate that Hg(II)_i_ bioavailability does not change significantly across the reservoir, and is thus unlikely to explain the trends in MeHg we observed. Overall, MeHg concentrations were correlated to the number of days since dissolved oxygen dropped below 0.5 mg/L (“days of anoxia”) in individual water samples (adjusted *R*^2^ = 0.32; *p* < 0.001), a correlation that improved by including year of sampling as a blocking factor (adjusted *R*^2^ = 0.43; *p* < 0.001; Fig. S15). Together, this suggests that MeHg concentrations are governed by on-going microbial Hg methylation activity in anoxic water.

### Microbial metabolic potential

Assembled shotgun metagenomes (Tables S2, S3) from the water column of Brownlee Reservoir were searched for metabolic genes to confirm the presence of TEAPs suggested by the geochemical data. Gene abundance was normalized to the median abundance of 16 universally conserved single-copy genes [47]; thus, the normalized gene abundance is presented as a percentage of the total microbial community. The abundance of other identified genes is provided in Table S4. Nitrate reductase genes (*narGHI*) were present at low levels (~ 10% of the microbial community) in oxygenated waters (Fig. S16; only *narG* is shown). Under oxygen-depleted conditions, *narG* increased in abundance, up to 50–60% at some depths, coincident with decreasing nitrate levels and increases in MeHg concentrations (Figs. S16, 2). Under sulfidic conditions at the bottom of RM286 in 2017, the abundance of *narG* decreased again. The genes enabling Mn-reduction via external electron transfer (EET) are highly diverse and poorly constrained [54, 76], making it difficult to use metagenomics to identify potential Mn reduction hotspots. However, homologs of *extE*, which is involved in Mn-reduction [77], were most abundant at 50 m at RM300 in 2019, coincident with a peak in filter-passing Mn (Table S4). These and other EET genes found in *Geobacter* were not present in the metagenomes from fall 2017 and 2018, but this does not rule out the presence of other Mn-reducing genes. We also searched for reductive *dsrA* and *dsrD* genes as markers for sulfate reduction (*dsrA* is shown in Fig. S16). In 2017, *dsrA* was present up to 4% in the deep hypolimnion at RM286, coincident with the detection of sulfide, but only to 0.5% at RM300, where nitrate was depleted but sulfide had not yet accumulated (Figs. S16, 2). In 2018, *dsrA* was most abundant (0.6%) under sulfidic conditions (RM300 at 47 m), but also present under nitrate-depleted conditions (0.2%). When nitrate was above detection, *dsrA* was low abundance (< 0.1%) except once (RM300 at 47 m in 2017), when nitrate was only 0.18 mg_N_/L and *dsrA* had an abundance of 0.27%. Thus, agreement was observed between *dsrA* abundance and geochemical indicators of dissimilatory sulfate reduction (sulfide, thiosulfate). Methanogenic-associated *mcrA* genes were only detected in 2017 and mostly found under sulfidic conditions (e.g., up to 2% abundance at the bottom of RM286) (Fig. S16). Overall, methanogenesis does not appear to be a prominent process occurring in the water column. These functional gene analyses (1) confirm that nitrate reduction was likely the dominant TEAP under anoxic conditions throughout Brownlee Reservoir across all study years and (2) provide modest evidence of dissimilatory sulfate reduction under nitrate-depleted conditions, when geochemical indicators of sulfate reduction were not yet detected.

### *hgcA* abundance

**Fig. 4:**
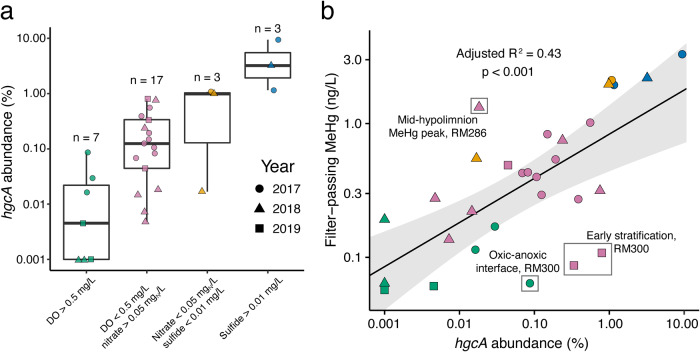
Methylmercury (MeHg) levels in Brownlee Reservoir are linked to abundance of *hgcA* gene. Abundance of *hgcA* by redox status (**a**) and a scatterplot of filter-passing MeHg concentrations against *hgcA* abundance (**b**). For both plots, the color of each point corresponds to the previously calculated redox status for that sample (Table S1), which is described on the x-axis of (**a**). Shape represents year of sampling. Black line in (**b**) represents the linear regression line, and the gray shaded area indicates the 95% confidence interval of the linear regression fit. Data were log-transformed before regression and both axes are presented on a log scale. Text on (**b**) explains origins of outlier points in gray boxes. Outliers were not statistically defined, and all points were included in the linear regression fit.

Assembled metagenomes were then searched for the *hgcA* gene and 26 unique *hgcA* genes were identified (Table S5). Total *hgcA* abundance was low in oxic waters (maximum = 0.09%; mean = 0.03 ± 0.02%; *n* = 7) and higher under oxygen-depleted conditions (maximum = 0.80%; mean = 0.23 ± 0.26%; *n* = 17; Fig. 4a). Total *hgcA* abundance was notably higher under sulfidic conditions (maximum = 9.4%; mean = 4.6 ± 4.3%; *n* = 3) than nitrate-depleted/non-sulfidic conditions (maximum = 1.1%; mean = 0.7 ± 0.6%; *n* = 3; Fig. 4a), despite comparable MeHg levels (Fig. 3). While direct comparisons of gene abundances across studies can be difficult due to different normalization methods, the relative abundance of *hgcA* under sulfidic conditions is comparable to that observed elsewhere in sulfidic lakes [26, 27] or highly reduced peat sediments [75], while *hgcA* abundance under nitrate-reducing conditions in Brownlee Reservoir is more comparable to that observed in marine waters [29, 78]. Across all years, there was a significant linear correlation (*p* < 0.001, *R*^2^ = 0.43) between *hgcA* abundance and filter-passing MeHg concentrations (Fig. 4b). While such a correlation seems intuitive, this relationship has not been observed in many environments, possibly due to factors such as changes in Hg(II)_i_ bioavailability, MeHg sinks (e.g., biotic or abiotic demethylation), or differences in transcription, translation, or protein activity of *hgcA* [78–84]. The outliers highlighted in Fig. 4b likely represent locations where these other factors substantially influence MeHg accumulation. Samples with more MeHg than expected based on *hgcA* abundance (above the regression line) may have had additional sources of MeHg (e.g., interflow transport of MeHg), while those with less MeHg than expected (below the regression line) may have been strongly influenced by a MeHg sink (e.g., demethylation) or located at a redox transition where MeHg concentrations are expected to lag behind *hgcA* abundance. Generally, the nitrate-depleted/sulfidic samples were above the regression line, possibly indicating that *hgcA*-containing organisms at this site were more efficient MeHg-producers, which is consistent with the observation in cultures that SRBs are particularly effective methylators [23]. Overall, the correlation between MeHg concentration and *hgcA* abundance in Brownlee Reservoir is consistent with geochemical profiles in suggesting water column Hg methylation as the primary source of MeHg. While this study used gene abundance rather than gene transcripts or protein abundance, the results are consistent with other studies that have shown a direct correlation between *hgcA* gene abundance and microbial Hg methylation by accounting for [85, 86] or controlling [75] the effects of Hg(II)_i_ bioavailability. In this river-reservoir system, the absence of strong geochemical gradients controlling Hg(II)_i_ bioavailability across the system (e.g., DOM chemistry, sulfide) suggest that MeHg production is primarily controlled by microbial Hg methylation capacity, here represented by the abundance of the *hgcA* gene.

### Metabolic capacity of Hg-methylating organisms

We generated metagenome-assembled genomes (MAGs) carrying *hgcA* (hgcA+) and predicted their metabolic capacity based on their gene content. We grouped these MAGs into ten unique, medium-quality (completeness>50%, redundancy<10% [87]) bacterial mOTUs. We assigned each mOTU to one of three functional guilds: obligately fermentative bacteria (FERM), high-redox respiratory organism (HRRO), or SRB. A brief description of each group follows and complete analyses of each mOTU are in the Supporting Information and Table S6.

#### Fermentative bacteria

**Fig. 5:**
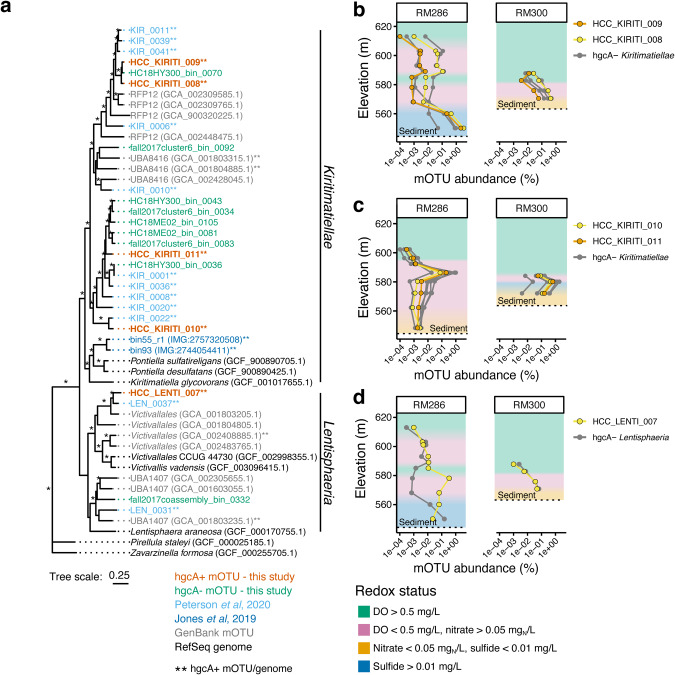
*Kiritimatiellae* and *Lentisphaeria* are abundant *hgcA*-carrying microbes in Brownlee Reservoir. Phylogeny (**a**) and depth distribution (**b**) of *Kiritimatiellae* (KIRITI) and *Lentisphaeria* (LENTI) mOTUs. Phylogenetic tree of hgcA+ and hgcA- mOTUs from this study (**a**). Tree includes reference genomes from RefSeq and reference mOTUs from Peterson et al. 2020, Jones et al. 2019, and from GenBank. An asterisk on a branch indicates >50% bootstrap support. Double asterisks after the mOTU or genome name indicate the presence of *hgcA*. Tree is rooted by two *Planctomycetota* genomes (*Pirellula staleyi* and *Zavarzinella formosa*). Abundance of two hgcA+ and several hgcA- *Kiritimatiallae* bins at RM286 and RM300 in 2017 (**b**) and 2018 (**c**). Abundan**c**e of hgcA+ and hgcA- *Lentisphaeria* at RM286 and RM300 in 2017 (**d**). The abundance values for the mOTUs on the x-axis for (**b**) through (**d**) are presented on a log scale. Shading behind the profiles in (**b**) through (**d**) represents the previously calculated redox status (Table S1).

The most common hgcA+ mOTUs across all redox conditions were designated as FERM, as indicated by the lack of terminal oxidases, electron transport chains, or other genes indicative of respiration. Five FERM mOTUs were phylum *Verrucomicrobiota*, four from class *Kiritimatiellae* and one from class *Lentisphaeria* (Fig. 5a, S17; Table S6). The *Kiritimatiellae* mOTUs from different years are phylogenetically distinct, but both cluster with hgcA+ mOTUs from the anoxic hypolimnion of a eutrophic freshwater lake [26] (Fig. 5a). Three of them were adapted to degrading polysaccharides, containing between 85 and 240 glycoside hydrolases (GHs; Table S6). They were most abundant in sulfidic waters (Fig. 5b, c). Eight additional *Kiritimatiellae* mOTUs without *hgcA* were closely related to the hgcA+ mOTUs (Fig. 5a) and showed similar abundance patterns (Fig. 5b, c). The *Lentisphaeria* mOTU with *hgcA* was most abundant in the upper hypolimnion at RM286 when nitrate levels were ~0.6 mg/L (Fig. 5d). This mOTU encoded an anaerobic sulfite reductase homolog and a *cydAB* terminal oxidase that could detoxify oxidants such as nitrite, sulfite, or oxygen [88], without gaining energy through respiration. *Kiritimatiellae* and *Lentisphaeria* organisms carrying *hgcA* are not confirmed Hg-methylators, but have now been shown to be abundant in several different aquatic ecosystems [26, 27, 86, 89]. An mOTU representing an obligately fermentative, polysaccharide-degrading *Firmicutes* organism was also retrieved from 2017 (Table S6).

#### High-redox respiratory organisms

**Fig. 6:**
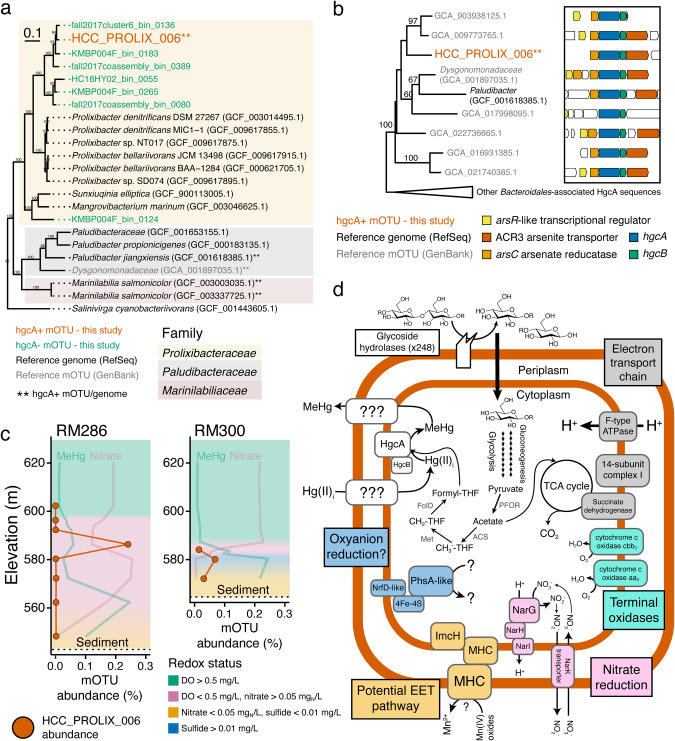
*Prolixibacteraceae* organism may link nitrate reduction to MeHg production in Brownlee Reservoir. HCC_PROLIX_006 mOTU phylogeny (**a**), HgcA phylogeny and *hgcA* gene neighborhood (**b**), abundance (**c**), and metabolic potential (**d**). Phylogenetic tree of HCC_PROLIX_006 within *Bacteroidales* using hgcA- mOTUs from this study, reference genomes from RefSeq, and one hgcA+ mOTU from GenBank (**a**). Tree was rooted using a genome from the *Salinivirgaceae* family, also within the *Bacteroidales* order. Genome/mOTU names followed by two asterisks indicates that it carries *hgcA*. Phylogenetic tree of HgcA sequences from HCC_PROLIX_006 compared to the other seven HgcA sequences from *Prolixibacteraceae* reference mOTUs and two additional closely related HgcA genes (**b**). Only bootstrap values above 50 are shown. Gene neighborhood annotations are based on kofamscan hits and subsequent validation with BLAST. Abundance of HCC_PROLIX_006 at RM286 and RM300 in 2018 (**c**). mOTU abundance is expressed as the average read coverage across the mOTU relative to the median read coverage of 16 ribosomal protein genes. Transparent profiles in background show MeHg and nitrate throughout the water column (no x-axis scale; see Fig. S5 for scale) and the background shading shows the calculated redox status of the water column (Table S1). Metabolic reconstruction of HCC_PROLIX_006 based on gene content (**d**). Metabolic predictions are based on converging approaches to annotate genes (see methods). See SI for detailed discussion of HCC_PROLIX_006 predicted metabolic capacity. Abbreviations: THF – tetrahydrofolate; PFOR – pyruvate-ferredoxin oxidoreductase; ACS – acetyl-CoA synthetase; FolD - 5,10-methenyl-H4F cyclohydrolase/5,10-methylene-H4F dehydrogenase; Met - 5,10-methylene-H4F reductase; Phs - Thiosulfate reductase; NrfD – membrane subunit of formate-dependent nitrite reductase; 4Fe-4S – iron-sulfur protein; MHC – multiheme cytochrome c; NarGHI – nitrate reductase; NarK – nitrate:nitrite transporter.

Two bacterial mOTUs were recovered that were predicted to be high-redox respiratory organisms (HRROs), capable of respiration using nitrate, Mn, or other unknown TEAs. One was classified into the *Prolixibacteraceae* family within the *Bacteroidales* order and designated HCC_PROLIX_006 (Fig. 6a). Six closely related medium-quality mOTUs without the *hgcA* gene were recovered (Fig. 6a). Of the 134 genomes and mOTUs in NCBI’s GenBank and RefSeq databases annotated as *Prolixibacteraceae*, only seven contained *hgcA*, none of which have been cultured (Figs. S18, 6b). The *Prolixibacteraceae hgcAB* gene pairs were co-located with several arsenic cycling genes, as observed in other hgcA+ organisms [25, 90, 91], which suggests a possible link between the biogeochemical cycling of these two elements. HCC_PROLIX_006 was recovered from the 2018 metagenomes and was most abundant in the metalimnion where MeHg was at a local maximum and nitrate was at a local minimum (Fig. 6c), suggesting that the organism was adapted to oxygen-depleted conditions. Correspondingly, HCC_PROLIX_006 contained a membrane-bound nitrate reductase and a full electron transport chain but lacked the genes for complete denitrification, instead carrying a *narK* gene encoding a nitrate importer/nitrite exporter (Fig. 6d). HCC_PROLIX_006 also encoded several possible pathways for EET, which could support Mn reduction. The mOTU also contained 248 glucoside hydrolyzes and complete central carbon metabolism pathways, suggesting that the organism can hydrolyze large polymeric carbohydrates and completely oxidize the resulting monomers. *Bacteroidetes* mOTUs carrying *hgcA*, closely related to HCC_PROLIX_006 but lacking *narG*, were present throughout the anoxic hypolimnion of a eutrophic lake [26]. Nitrate reduction genes have been identified in hgcA+ mOTUs [26, 29] but have not been directly implicated in MeHg production.

The second HRRO mOTU (designated HCC_PELOB_005) was classified as *Pelobacteraceae*, which is closely related to *Geobacteraceae* (Fig. S19a). HCC_PELOB_005 contained several genes encoding pathways for EET, including *extE* [77] and both *imcH* and *cbcL*, which facilitate EET to high- and low-redox TEAPs, respectively (Fig. S19b) [92]. HCC_PELOB_005 was most abundant at 50 m at RM300 in 2019 (Table S6). While particulate Mn was low at that site, filter-passing Mn was present. Mn oxides can cycle quickly at steep oxic gradients [68, 93], suggesting a possible cryptic Mn cycle linked to MeHg production. An hgcA+ *Pelobacteraceae* mOTU metabolically and phylogenetically similar to HCC_PELOB_005 was identified in a freshwater lake at the oxic-anoxic interface where Mn reduction was suspected (GEO_0030 in Fig. S19) [26]. While Mn respiration has not been shown to drive MeHg production, Fe-reducing *Geobacter*, closely related to *Pelobacteraceae*, are also capable of reducing Mn [92]; are known to be efficient Hg-methylators [23, 94]; and have been implicated in MeHg production in rice paddies [82] and lake sediments [95].

#### Sulfate-reducing Hg-methylators

We also recovered four mOTUs representing SRB. Three were classified in the *Desulfobacterales* order. These mOTUs each had at least partial reductive *dsr* operons and an electron transport chain. In 2017, these mOTUs were only present when sulfide was detected in the water column (Fig. S20). The fourth potential SRB mOTU was associated with the *Smithellaceae* family and recovered from 2019 metagenomes. While this mOTU contained *dsrABD*, it lacked other key genes for sulfate reduction and was detected at 50 and 56 m at RM300, despite concentrations of 1.4 mg_N_/L nitrate present, suggesting the mOTU may not be a true SRB. This lack of a complete sulfate-reducing pathway has been previously reported [96] and is consistent with previous work suggesting that Smithellaceae are primarily fermentative and occasionally syntrophic [97].

### Biogeochemical drivers of microbial Hg methylation

**Fig. 7:**
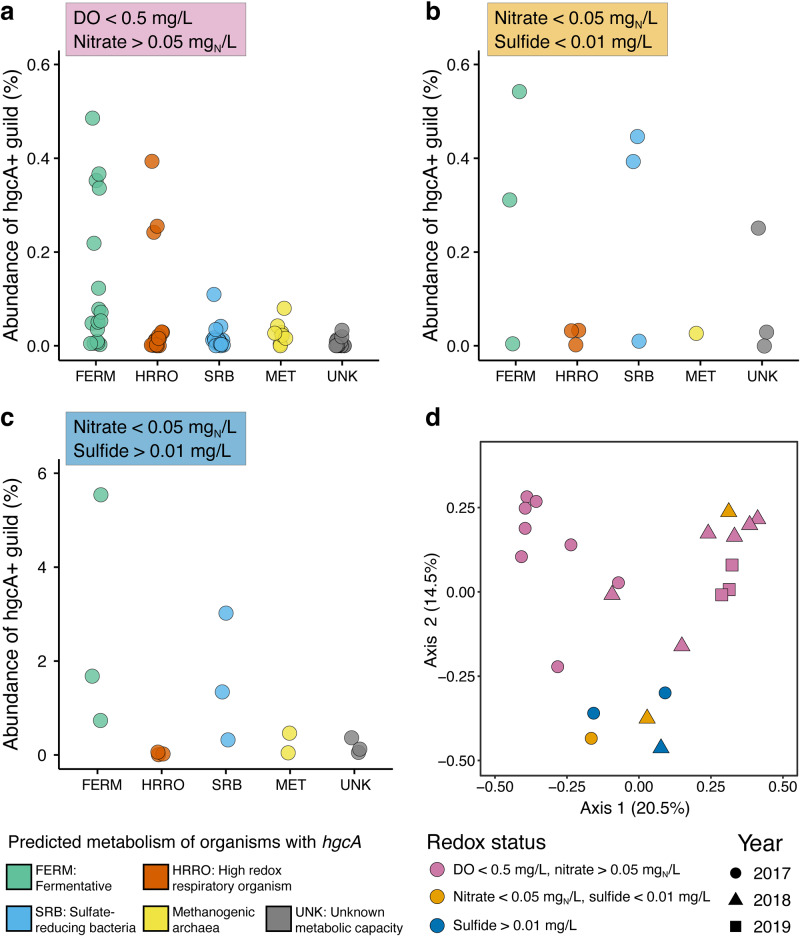
Metabolically diverse microbes carry * hgcA* genes under high and low redox conditions. Overall abundance of functional guilds of *hgcA*-carrying microbes in individual samples under oxygen-depleted (**a**), nitrate-depleted (**b**), and sulfidic conditions (**c**); and beta-diversity of *hgcA* genes in all samples (**d**). Read coverage of each *hgcA* gene was normalized to the read coverage of 16 ribosomal protein genes to calculate *hgcA* abundance. Each *hgcA* sequence was assigned a predicted metabolic guild. Each point in (**a**) through (**c**) corresponds to the total abundance in a single metagenome of the *hgcA* genes assigned to specific guild. Note the different scales on the y-axis for (**c**). For the ordination in (**d**), the *hgcA* population was assessed using nonmetric multidimensional scaling based on pairwise Bray-Curtis dissimilarity using normalized *hgcA* gene abundance. The percent values on the axis labels represent the percent variation explained by that coordination plane. For (**d**), Colors of the points represent the calculated redox state (Table S1), and the shapes represent the year the sample was collected.

To understand the relative contribution of different microbial guilds to the total *hgcA* population, each of the 26 metagenomic *hgcA* sequences was assigned a functional guild, either one that was used for the mOTUs (FERM, HRRO, or SRB) or methanogens (MET), which forms a distinct phylogenetic group with *Chloroflexi* within the HgcA phylogeny [25] (Table S5; Fig. S21). Sequences that could not be assigned a functional guild were designated unknown (UNK). In anoxic water with nitrate >0.05 mg/L, FERM were the dominant *hgcA*-containing microbes (> 60% of the total *hgcA* abundance) (Fig. 7a). HRROs with *hgcA* were low in abundance in most oxygen-depleted samples; however, at three such sites, hgcA+ HRROs were highly abundant, mostly represented by the two HRRO mOTUs described above (Fig. 7a). Nitrate-depleted conditions, regardless of sulfide presence, were dominated by FERM and SRB-associated *hgcA* sequences (Fig. 7b, c). MET-associated *hgcA* sequences were rare throughout the reservoir. A nonmetric multidimensional scaling ordination based on Bray-Curtis dissimilarity confirmed that the hgcA+ microbial communities under nitrate-depleted but non-sulfidic conditions were similar to those under sulfidic conditions, while the hgcA+ microbial community from oxygen-depleted samples was distinct (Fig. 7d).

**Fig. 8:**
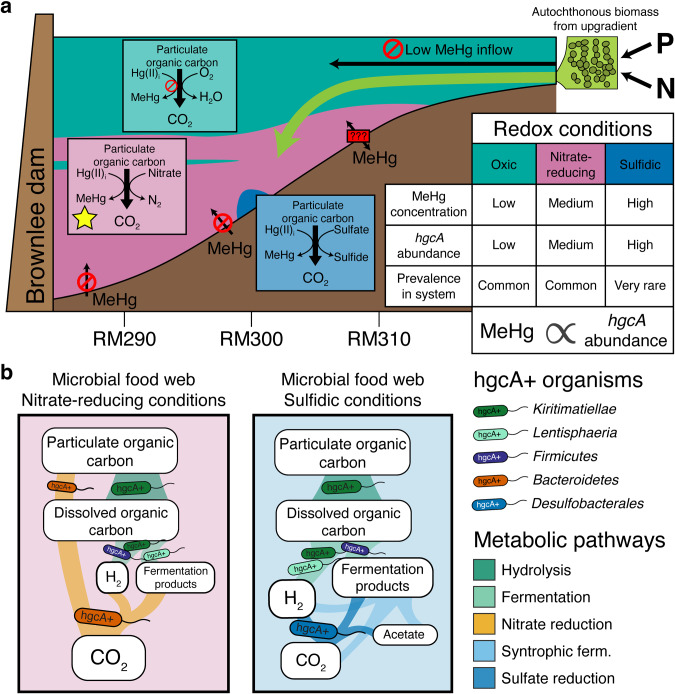
Coupled metagenomic-biogeochemical approach improves conceptual understanding of Hg-cycling in Brownlee Reservoir. Conceptual model of drivers of MeHg production in Brownlee Reservoir on the macro (**a**) and micro scale (**b**). (**a**) Oxygen-depleted conditions are the consequence of high levels of autochthonous organic carbon from the Snake River, which is in turn the result of agricultural run-off rich in nitrogen and phosphorus. Hypolimnetic accumulation of MeHg cannot be accounted for by inflowing water or by MeHg diffusion from the sediments, except in the transition zone upgradient of RM308. Thus, MeHg accumulation is dominated by production within the water column. Although MeHg and *hgcA* are highest under sulfidic conditions, these are rare throughout the reservoir. Nitrate-reducing conditions (yellow star) are common throughout Brownlee Reservoir and promote moderate MeHg accumulation. **(b)** Under nitrate-reducing conditions, particulate organic carbon can either be hydrolyzed and fermented by fermentative organisms, supplying respiratory organisms with fermentation end-products, or be completely oxidized by nitrate-reducing organisms. Bacteria with *hgcA* were identified at each step of this microbial food web. Under sulfidic conditions, particulate organic carbon is hydrolyzed and fermented by a consortium of fermentative and syntrophic organisms whose metabolism is linked to the activity of sulfate-reducing bacteria. Fermentative and sulfate-reducing bacteria with *hgcA* were both identified in Brownlee Reservoir under sulfidic conditions.

The wide range of metabolic functions associated with hgcA+ microbes in Brownlee Reservoir points to a conceptual model in which multiple biogeochemical cycles influence microbial MeHg production capacity rather than just one or two (Fig. 8). The relative contribution of hgcA+ organisms from different metabolic guilds to overall MeHg production is unknown. Environmental metatranscriptomic studies show different *hgcA* expression levels from different microbial guilds [25, 98] and laboratory studies show variable MeHg production rates between hgcA+ microorganisms [16, 23, 99]. This reinforces the need to pair *hgcA* gene abundance data with relevant geochemical analysis to identify potential biogeochemical drivers and to interpret that data carefully. Here, paired water chemistry and metagenomic data support that microbial nitrate reduction is far more likely than sulfate reduction to fuel most MeHg production (Fig. 8). This is of particular concern in Brownlee Reservoir, where long-term monitoring identified a nearly two-fold increase in inflowing nitrate concentrations from 1995 to 2021 [39]. However, the predominance of fermentative hgcA+ organisms (Fig. 7) and the correlation between MeHg and days of anoxia (Fig. S15) also points to overall carbon metabolism under anoxic conditions as a major driver of microbial MeHg production, a link previously documented in lacustrine water columns [67] and wetland periphyton [100]. Most of the organic carbon in this system is autochthonous cyanobacterial or algal biomass produced within the Snake River and/or Brownlee Reservoir [6], driven by large nitrogen and phosphorus inputs from agricultural runoff [101] (Fig. 8). Under the nitrate-replete conditions common throughout Brownlee Reservoir, particulate organic matter can be hydrolyzed and fermented by obligately fermentative organisms that provide simplified organic acids to HRROs for oxidation through nitrate- or Mn/Fe-reduction; alternatively, it can be hydrolyzed and oxidized solely by HRROs with complete central carbon metabolism pathways. In the small pockets of sulfidic water, SRBs rely on fermentative organisms to provide them with small organic acids or monomeric compounds [102]. Microbial community metabolism is often limited by the initial hydrolysis of complex organic carbon molecules [103], so regardless of the functional guild producing MeHg, the supply and catabolism of labile carbon is likely the limiting factor for microbial Hg-methylation activity. This suggests that more than any given TEAP, overall carbon metabolism and polymeric hydrolysis under anoxic conditions is the primary biogeochemical process linked to microbial Hg-methylation activity. If so, the Snake River-Hells Canyon Total Maximum Daily Load (TMDL) aimed at reducing algae levels and improving DO conditions, if successful, has the potential to reduce internal MeHg loading in Brownlee Reservoir [39, 104]. While the tools to directly quantify the contribution of various functional groups to MeHg production do not exist at this time, the functional diversity of hgcA+ organisms and the possible link to overall metabolism suggest that quantifying the relationship between microbial Hg-methylation activity and metabolic processes and confirming the rate-limiting steps of microbial metabolism (i.e., polymer hydrolysis vs. TEAP rates) will provide insight into the dominant biogeochemical factors driving MeHg production.

### Environmental implications

Reservoirs are dynamic systems that can have significant impacts on Hg accumulation in aquatic food webs within and downstream of impounded waters [5, 9]. In Brownlee Reservoir, hydrologic conditions determined the location of the deposition zone and subsequent progression of anoxia and redox changes. Interannual differences in river-reservoir hydrology had cascading effects on the water chemistry, progression of TEAPs, and abundance and metabolic potential of Hg-methylating organisms, all of which ultimately modulated differences in MeHg concentrations in Brownlee Reservoir. The observed variation in location of MeHg production is likely to translate to spatial differences in MeHg bioaccumulation in the food web and temporal differences in MeHg mixing into the interflow and subsequent export through the dam [8, 36]. Understanding the coupled hydrologic and biogeochemical drivers of Hg methylation is important to developing management strategies to limit MeHg formation and bioaccumulation. The assumption that MeHg production is primarily mediated by SRB and methanogens has led to efforts to reduce MeHg contamination of aquatic food webs by hypolimnetic nitrate amendment [18] or Mn oxides [20, 21] as a cost-effective alternative to hypolimnetic oxygenation [17]. However, the results presented here suggest that MeHg production can occur under denitrifying or Mn-reducing conditions by HRROs or their fermentative partners. Thus, while hypolimnetic amendments have been effective in some systems [18, 20, 21], caution should be used in deploying these strategies. Comprehensive studies and/or trial experiments identifying potential microbial factors linking organic carbon metabolism and TEA concentrations to MeHg production are critical to verify the efficacy of chemical amendments on MeHg production [20]. The deployment of robust experimental designs and new ecophysiology methods will permit the investigation of diverse microbial functional guilds and their contribution to MeHg production as a function of major biogeochemical cycles and net productivity of waterbodies; this will subsequently permit targeted management strategies to mitigate MeHg uptake in aquatic food webs and the risk of Hg to humans.

## Supplementary information

Supplemental Information

Supplemental Tables

## Data Availability

The water chemistry data analyzed in the current study are available as a U.S. Geological Survey data release (10.5066/P9DT2B6J). Raw metagenomic sequencing data are stored in the NCBI database under BioProject PRJNA878929. Processed sequencing data, including HgcA amino acid sequences, *hgcA* gene sequences, and genomic bins containing the *hgcA* gene are available online at FigShare under project #158018. Code used to generate figures and analyze data is available on Github (https://github.com/petersonben50/HellsCanyon).
