# Supplementary material for: Metabolically diverse microorganisms mediate methylmercury formation under nitrate-reducing conditions in a dynamic hydroelectric reservoir
Source: ISME J. 2023 Jul 26;17(10):1705–18. doi: 10.1038/s41396-023-01482-1 (PMC10504345; doi:10.1038/s41396-023-01482-1)
Supplement: Supplementary file 1 — Supplemental Information [file 41396_2023_1482_MOESM1_ESM.pdf]

Supplementary Information: Metabolically diverse microorganisms mediate methylmercury formation under nitrate-reducing conditions in dynamic hydroelectric reservoir

Benjamin D. Peterson[1,2,3]*, Brett A. Poulin[3], David P. Krabbenhoft[4], Michael T. Tate[4], Austin K. Baldwin[5], Jesse Naymik[6], Nick Gastelecutto[6], Katherine D. McMahon[1,2]

1. Department of Civil and Environmental Engineering, University of Wisconsin - Madison, Madison, Wisconsin 53706, United States
2. Department of Bacteriology, University of Wisconsin - Madison, Madison, WI 53706, United States
3. Department of Environmental Toxicology, University of California - Davis, Davis, CA 95616, United States
4. U.S. Geological Survey, Upper Midwest Water Science Center, Mercury Research Laboratory, Madison, Wisconsin 53726, United States
5. U.S. Geological Survey, Idaho Water Science Center, Boise, Idaho 83702, United States
6. Idaho Power Company, Boise, Idaho 83702, United States

*Corresponding author: bdpeterson@ucdavis.edu

Supplementary Information includes 21 figures and 54 pages.

Supplementary tables are contained in separate Excel file.

Any use of trade, firm, or product names is for descriptive purposes only and does not imply endorsement by the U.S. Government.

Table of contents

Supplemental Materials and Methods: 3

Field site and sampling 3

Water chemistry analyses 6

DNA extractions 8

Metagenomic sequencing and processing 9

hgcA analysis 12

Statistical analyses 14

Supplementary Results and Discussion 15

Fermentative hgcA+ mOTUs 15

High-redox respiratory hgcA+ mOTUs 17

Sulfate-reducing hgcA+ mOTUs 20

References 22

Supplemental Tables 32

Supplementary Figures 33

Supplemental Materials and Methods:

Field site and sampling

Field sites: This study includes laboratory analyses of surface water and porewater samples collected from locations within Brownlee Reservoir of the Hells Canyon Complex (Idaho, Oregon) of the Snake River, from 2016 to 2019. The reservoir spans approximately 61 river miles of the Snake River and has a hydraulic retention time of 34 days. Sampling locations included: one site on the main stem of the Snake River upstream of Brownlee Reservoir (Snake River Mile (RM) 345.6) that is designated as the inflow to the reservoir; three sites within Brownlee Reservoir (RM310, RM300, and RM286; the data release also contains data for RM318, RM314, and RM305 that is not presented in this manuscript); and one reservoir outflow location (RM283.9, at the Brownlee Reservoir dam). A parallel study investigating the long-term response of dissolved oxygen (DO) to inflowing nutrients and organic matter (Naymik et al., 2023) split the study area into three zones: riverine, transition, and lacustrine (Thornton, 1990). RM310 is within the transition zone, while RM286 and RM300 are in the lacustrine zone.

Sampling methods: All water samples were collected using ultraclean trace metal methods. Sample containers were triple rinsed with site water before collection. *Inflow/outflow sampling*: At the Brownlee Reservoir inflow and outflow locations, whole water and filtered surface water samples were collected from a bridge using a depth-integrated sampler. Inflow samples in 2016 and 2017 were collected from the side of the river, due to bridge construction, using a polyethylene terephthalate (PETG) bottle for a grab sample (further details provided in the metadata file of the data release). *In-reservoir water column sampling*: At the sites within Brownlee Reservoir, whole water and filtered water samples were collected from a boat in-profile from the surface (2 meters) to approximately 5 meters above the sediment-water interface

using one of two methods. 1. A large-diameter Teflon tube was equipped with acid-washed C-Flex tubing and a peristaltic pump was used to pump water up from sampling depth. 30 L of water was wasted through the line before sample collection. Filtered samples (dissolved organic carbon (DOC) concentration, dissolved organic matter (DOM) characterization, anions, sulfide, and filter-passing cations) were filtered in-line using a 0.45 μm GeoTech Versapor capsule filter directly into the container and preserved (see below). Unfiltered samples were collected directly the sampling container and preserved (see below). 2. An acid-washed, trace-metal clean Van Dorn sampler was also used to collect samples from the water column. Filtered samples (DOC, DOM characterization, anions, and filter-passing cations) were field-filtered with a syringe and a 0.45 μm Whatman polyethersulfone (PES) disk filter directly into the collection bottle and preserved (see below). Unfiltered samples were poured directly the sampling container and preserved (see below). For both methods, up to two replicate samples were collected per profile. Multiparameter sonde profiles were collected concurrent to water sampling, which included dissolved oxygen concentration, oxidation-reduction potential, pH, specific conductance, and turbidity. *In-reservoir sediment:water interface sampling*: Undisturbed sediment cores were collected to a depth of approximately 0.1 m with a HTH sediment corer with a 70 mm outside diameter. This left up to 1 m of water intact above the sediment:water interface within the core barrel. Water above the sediment-water interface (termed "corewater") was obtained by collecting water in vertical subsections core using ¼" Teflon tubing attached to acid-washed C-Flex tubing and a peristaltic pump. Water was collected into a PETG bottle and homogenized before allocation into sampling containers. Unfiltered samples (for Hg and unfiltered cations) were poured into the sampling container and preserved (see below). Filtered samples (DOC, anions, filtered cations, and sulfide) were field-filtered with a syringe and a 0.45 μm Whatman

PES disk filter directly into the collection bottle and preserved (see below). *Porewater sampling*: Porewater samples were collected from the top five cm of sediment cores, collected independently of the cores used for corewater sampling. *Sonde profiles*: Profiles of temperature and DO were collected using an SBE 19plus SeaCAT Profiler CTD (SeaBird Scientific) along the thalweg of Brownlee Reservoir every two river miles approximately twice monthly from 2016 – 2019 (additional years and parameters included in the data release).

Sample preservation: Unfiltered water was collected for Hg speciation analysis into a 2L PETG bottle (125 ml PETG for corewater and porewater samples) and stored with no headspace in the dark. Samples were vacuum-filtered in the lab with a quartz fiber filter (QFF, nominal pore size 0.7 µm) and acidified to 1% HCl for filter-passing MeHg and total Hg analysis. QFFs containing particulate matter were frozen and stored at -20˚C for particulate Hg analysis. Samples collected for filtered and unfiltered metals/cations were preserved to 1% nitric acid. Samples for anion analysis (nitrate, sulfide, and thiosulfate are presented in this study) were frozen on dry ice in the field then stored at -20˚C until analysis. Filtered samples for DOC concentration and DOM characterization were stored on ice in the dark until they could be moved to a refrigerator and analyzed within a month of collection. Samples for inorganic sulfide were preserved with 50% sulfide anti-oxidant buffer and analyzed within 24 hours. Microbial samples for metagenomic sequencing were collected onto 0.22 µm Sterivex filters and flash frozen on liquid nitrogen within 60 seconds. Sterivex filters were then transported back to the lab on dry ice or liquid nitrogen and stored at -80˚C.

Water chemistry analyses

Hg analyses: All Hg samples were analyzed at the U.S. Geological Survey Mercury Research Lab (MRL). Complete analytical methods and QC parameters are described in detail here: https://wi.water.usgs.gov/mercury-lab/research/analysis-methods.html. All analyses conformed to MRL established QC checks, including: <10% deviation/RSD for technical replicates, 90-110% for continuing calibration checks, and matrix spike recovery between 90 and 110%. The detection limit is 0.04 ng/L for aqueous total Hg (HgT) and 0.01 ng/L for methylmercury (MeHg). Aqueous and particulate HgT determination followed U.S. Environmental Protection Agency (EPA) Method 1631, revision E (U.S. EPA, 2002) and the USGS Techniques and Methods 5 A-8 (Olund et al., 2004), respectively. Briefly, this consists of sample oxidation with bromine monochloride (BrCl), neutralization of BrCl with hydroxylamine, tin chloride reduction, purge and trap with dual gold trap amalgamation, and quantification by cold vapor atomic fluorescence spectrometry. Filter-passing and particulate MeHg were analyzed following a modified version of EPA method 1630 and passed all required quality assurance and control objectives as stated by the USGS MRL. The modified method includes sample preparation with the Brooks Rand Merx-M and detection with inductively coupled plasma mass spectrometry (ICP-MS), with quantification by isotope dilution (DeWild et al., 2002; Horvat et al., 1993; Lepak et al., 2015). Samples were spiked with $Me^{199}Hg$, amended with copper sulfate (CuSO4) (for aqueous samples) or a dilute CuSO4/sulfuric acid/potassium chloride solution (for filters), and distilled under nitrogen gas at 121℃. Sodium tetraethylborate was added to the distillate to ethylate Hg species. The ethylated Hg was purged with argon, captured on Tenax traps, thermally desorbed, separated isothermally by mass with gas chromatography (all on the Merx-M), and introduced to the ICP-MS (Thermo Fisher Scientific). Concentrations of

particulate and filter-passing inorganic divalent Hg ($Hg(II)_i$)were calculated by subtracting particulate MeHg and filter-passing MeHg concentrations from particulate HgT and filter-passing HgT concentrations, respectively.

Cations: Unfiltered acidified samples for cation analysis were digested in 3% nitric acid, heated to 65˚C for 8 hours, and filtered with a 0.45 µm PES syringe filter (Garbarino & Hoffman, 1999) then analyzed using inductively coupled plasma-optical emission spectrometry (ICP-OES; Optima 5300 DV, Perkin Elmer). Filter-passing cations were analyzed by ICP-OES (Optima 5300 DV, Perkin Elmer) or by ICP-MS (NexION 300Q, Perkin Elmer). Particulate Fe and Mn concentrations were calculated as the difference between concentration in the unfiltered and filter-passing samples.

Anions: Anions (nitrate, sulfate, thiosulfate) were analyzed by ion chromatography (Thermo Scientific, Dionex DX120). Frozen samples were thawed within 20 minutes of analysis on the IC. Thiosulfate samples were only measured on samples that were immediately frozen upon collection.

Sulfide: Sulfide was analyzed within 12 hours of sample collection using an Orion silver/sulfide ion selective electrode.

Carbon: DOC was quantified by persulfate oxidation on a total organic carbon analyzer (Ocean Instruments Model 700). The UV-vis absorbance spectra were measured on a spectrophotometer (Agilent HP8453 ultraviolet-visible spectrophotometer) from 190-800 nm. The absorption coefficient at 254 nm was divided by the DOC concentration to obtain the specific UV absorbance at 254 nm ($SUVA_{254}$, quantified in units of L/mg carbon/m) (Weishaar et al., 2003).

DNA extractions

DNA was extracted using a modified protocol involving enzymatic and physical cell disruption, phenol-chloroform extraction, and purification by isopropanol/ethanol precipitation (Lever et al., 2015; Peterson et al., 2020). Sterivex filter cartridges were cut open using a flame-sterilized PVC cutter and the filter removed using flame-sterilized razor blades and forceps. Half of the filter was placed into a bead-beating tube with Lysing Matrix A (MP Biomedical), which consists of a zirconium sphere and garnet flakes. Cells were lysed by addition of 250 μL of 1mg/mL lysozyme with 1 unit of RNase A (ThermoFisher Scientific) and incubation for 10 min at room temperature. Proteins were digested with 250 μL of 1mg/mL proteinase K in a 2XS buffer (2% xanthogenate, 40mM EDTA, 1.6M ammonium acetate, 2% SDS, in a 200mM Tris buffer, pH7.5) for 3 min at room temperature. Cells were physically disrupted by 90 seconds of beadbeating. Polysaccharides were precipitated out by addition of 125 μL of 5M NaCl followed by 125 μL of 10% CTAB and incubation at 70℃ for 20 min. DNA was then extracted twice with 750 μL of phenol:chloroform:isoamylalcohol that was buffered to pH=8.0 (25:24:1; Fisher BioReagents), followed by a single wash extraction with 750 μL of chloroform. Each extraction consisted of reagent addition, mixing for 3 min, centrifugation at 13200g for 5 min, and the transfer of the supernatant to a new 1.5 ml microcentrifuge tube. DNA was then precipitated by addition of 750 μL 100% isopropanol at -20˚C. DNA was precipitated at -20℃ overnight, then centrifuged at room temperature at 13200g for 15 min. The supernatant was carefully removed and the DNA pellet washed with 1 mL of 70% ethanol, then centrifuged for another 15 min at 13200g. The supernatant was removed and the DNA dried on the bench. When dry, the DNA was resuspended in 50 μL of TE buffer. DNA was then further purified using a QIAquick PCR

purification column, following the manufacturer's instructions. DNA was quantified using a Qubit Fluorometer and quality was assessed on a Nanodrop.

Metagenomic sequencing and processing

500 ng of extracted DNA was sent to the Functional Genomics Lab (QB3, Berkeley, CA). DNA was sheered and sized-selected for 600 bp sequences. Kapa Biosystem Library Prep kit (Roche Sequencing and Life Science, Kapa Biosystems, Wilmington, MA) was used to generate sequencing libraries. Sequencing was completed at the Vincent J. Coates Genomics Sequencing Lab (QB3, Berkeley, CA). The 2018 and 2019 metagenomes were generated on an Illumina NovaSeq using the S4 chemistry, while 2017 samples were generated on an Illumina HiSeq4000. All metagenomes consisted of 150 bp paired-end reads.

Bioinformatic processing was completed on the UW-Madison Great Lakes Bioenergy Research Center computing cluster. All bioinformatics scripts are available online at https://github.com/petersonben50/HellsCanyon. Metagenome read counts before trimming ranged from 16 million to 478 million paired-end reads, with a median of 55 million (Table S1). Ends of reads were quality trimmed (PHRED score < 20), short reads (<100 bp) were discarded, and overlapping read pairs were merged using fastp (v0.20.1) (Chen et al., 2018). More than 95% of the total read coverage was retained after trimming. Metagenomes were clustered by kmer-content using Mash (v2.2.2), with kmer length of 21 and sampling size of 100,000 kmers (Ondov et al., 2016). Metagenomes from 2017 and 2018 were manually organized into clusters based on Mash similarity and coassembled within that cluster (Table S3). Metagenomes from 2019 were assembled individually due to the larger size of the metagenomes and the less similarity between them. All metagenomes from 2017 were also coassembled. The coassembly

of all metagenomes from 2018 was not completed due to memory constraints on the computing cluster. All assemblies were done using metaSPADes (v3.14.1) (Nurk et al., 2017). Assembly statistics are shown in Table S3. Contigs shorter than 1000 bp were discarded using anvi-script-reformat-fasta from anvi'o (Eren et al., 2015). Open reading frames (ORFs) were predicted using Prodigal (v2.6.3) (Hyatt et al., 2010) on metagenome mode. Reads were mapped back to the contigs using bowtie2 (v2.6.3) (Langmead & Salzberg, 2012). Coverage of each contig was calculated by calculating the median number of reads maps to each residue using samtools (v1.16.1) (Li et al., 2009). The 150 bp on either end were not included in this calculation to eliminate potential bias due to reduced mapping of reads overlapping the end of the contigs. The abundance of individual genes in the assemblies were assigned as the read coverage of the contig containing the gene. Abundance of bins/mOTUs was calculated as the median read coverage of the contigs contained within the mOTU. To normalize abundance across metagenomes, we calculated the coverage of 16 different ribosomal protein (rp16) genes in each assembly (Anantharaman et al., 2016; Sorek et al., 2007). The coverage of each gene and mOTU within a metagenome was normalized to the median coverage of the rp16 genes for that metagenome. Metabolic genes in the assemblies were initially identified using Hidden Markov Models (HMMs). Terminal electron accepting process (TEAP) genes that are presented in main text were confirmed using maximum-likelihood phylogenetic reconstruction of the amino acid sequences, similar to the method used in this manuscript for phylogenetic reconstruction of HgcA sequences. To identify reductive vs. oxidative *dsrA* genes, DsrA amino acid sequences were aligned to the dataset from Müller et al, 2015 where they identified phylogenetic conservation of reductive vs. oxidative DsrA proteins (Müller et al., 2015). A maximum-likelihood tree using FastTree (Price et al., 2010) was then generated and the DsrA sequences from this study were

classified as oxidative or reductive based on their phylogenetic placement.Automatic binning was done using Metabat2 (v2:2.15) (Kang et al., 2019) and MaxBin2 (v2.2.7) (Wu et al., 2016), which were then aggregated using Das Tool (v1.1.2) (Sieber et al., 2018). CONCOCT, implemented within anvi'o, was used to generate binned clusters of contigs based on tetranucleotide frequency and differential abundance. The number of clusters was constrained to approximately 1/2 to 1/3 of the number of expected genomes in the sample to minimize fragmentation error, based on previous work (Delmont et al., 2018). Clustered contigs an *hgcA* gene (hgcA+) were then binned manually curated in anvi'o (v6.2) (Eren et al., 2015) using the automatic binning results as a reference. Automatically and manually generated bins were both included in the final analysis and were dereplicated by grouping into mOTUs that shared 98% average nucleotide identity (ANI) and had 50% alignment fraction using the ANI Calculator (Varghese et al., 2015) as implemented by Sarah Stevens (https://github.com/sstevens2/ani_compare_dag). In all, we identified 16 medium-quality bins (> 50% completeness, < 10% redundant as defined by the MIMAG quality standards (Bowers et al., 2017)), carrying *hgcA* that were grouped into 10 metagenome operational taxonomic units (mOTUs). Information for each bin/mOTU is in Table S6. Phylogenetic reconstruction and metabolic pathway identification returned nearly identical results for each of the bins within a mOTU, so the results for one selected representative within each mOTU is described in this manuscript. Most phylogenetic clusters of *hgcA* included at least one binned *hgcA* sequence (Fig. S21). The taxonomy of each bin was estimated using GTDB-TK (Chaumeil et al., 2019). All taxonomic annotations are based on the GTDB taxonomy (D. H. Parks et al., 2022). Metabolic annotations of the bins were done using convergent approaches, including kofamscan (Aramaki et al., 2020), custom HMMs with hmmer (Eddy, 2015), METABOLIC (Zhou et al., 2022), and

FEET (Olmsted et al., 2022). Major TEAP gene annotations were confirmed by phylogenetic reconstruction. Phylogenetic trees of bins were based on alignments of the rp16 genes (Anantharaman et al., 2016). Genome references were identified from the GTDB tree generated through the GTDB-TK workflow, located in the National Center for Biotechnology Information (NCBI) RefSeq and GenBank databases, and downloaded using NCBI's Entrez (Sayers et al., 2021).

hgcA analysis

HgcA sequences were identified from the assembly-derived and mOTU-derived ORFs using the same method. A custom HMM based on HgcA amino acid sequences from experimentally confirmed Hg methylators (Peterson et al., 2020) was used to identify potential HgcA sequences. The trusted cut-off score of 132 was used, based on prior testing that showed this excluded HgcA homologs while including all complete HgcA sequences. Of the identified sequences, HgcA sequences without the cap helix domain N(V/I)WCA(A/G/S)GK (J. M. Parks et al., 2013) were removed. Truncated sequences without any predicted transmembrane domains at the C-terminal end were not included in the abundance calculations but are included in Table S5 for completeness. All manually confirmed *hgcA* genes were then clustered at 97% identity to dereplicate sequences across assemblies. In this manuscript, we discuss one representative from each cluster, chosen for its completeness, but the described analyses are congruent for most sequences within a cluster. 26 total unique *hgcA* genes were identified. Most (19 out of 26) *hgcA* genes had a *hgcB* gene immediately downstream. For four *hgcA* genes, the scaffold terminated immediately after the *hgcA*, suggesting the *hgcB* was simply not assembled into the contig. There were also three unique *hgcA* sequences that had ORFs downstream that were not *hgcB*. These

downstream ORFs could not be identified using NCBI BLAST or MOTIF (https://www.genome.jp/tools/motif/). Because it has been shown that the *hgcB* gene can be located elsewhere on the genome and the corresponding *hgcA* could still be functional (Goñi-Urriza et al., 2020; J. M. Parks et al., 2013; Ranchou-Peyruse et al., 2009), we kept these sequences in the analyses. HgcA amino acid sequences were aligned with MUSCLE (v3.8.31) (Edgar, 2004). That alignment was aligned to references from Hg-MATE (v1.01142021) (Gionfriddo et al., 2021). Residues with 50% gaps were masked. A maximum-likelihood tree was generated from this alignment using RAxML (v8.2.11) (Stamatakis, 2014) under the GAMMA distribution with the LG model. Automatic rapid bootstrapping was used to generate branch support (550 total bootstraps). The tree was then mid-point rooted using the phangorn package (v2.7.0) (Schliep, 2011) and visualized using ggtree (v3.1.1) (Yu et al., 2017) in R (v4.1.3). HgcA sequences were also classified using the Hg-MATE database with an established workflow (Gionfriddo, Wymore, et al., 2020; Gionfriddo et al., 2021) based on pplacer and guppy (Matsen et al., 2010). Using the taxonomy of hgcA+ mOTUs from this study, *hgcA* sequences from the Hg-MATE database, and hgcA+ mOTUs from several recently published papers (Jones et al., 2019; Peterson et al., 2020), each *hgcA* gene was manually assigned a taxonomy. However, it is important to note that prior work suggests that *hgcA* has likely undergone extensive horizontal gene transfer (HGT) (Gionfriddo, Wymore, et al., 2020; McDaniel et al., 2020; Podar et al., 2015) and thus taxonomic assignment of an organism based on this single gene should be interpreted with caution. However, HgcA phylogeny at the fine scale is often congruent with organismal taxonomy (McDaniel et al., 2020) and thus we feel confident in the broad trends we are describing using this approach. Each of these *hgcA*-carrying organisms was also assigned a metabolic function based on the metabolic reconstruction of

mOTUs with a closely related *hgcA* gene. We grouped sequences into one of four metabolic guilds: fermentative (FERM), when mOTUs were lacking in terminal oxidases, electron transport chains, and/or multi-heme cytochrome c genes; high-redox respiratory organisms (HRROs), which included sequences associated with mOTUs expected to reduce nitrate and/or Mn oxides; sulfate reducing bacteria (SRB), which carried the *dsr* gene cassette or other sulfate/sulfite-reducing machinery; and methanogens (MET). No methanogenic mOTUs carrying *hgcA* were identified, but the methanogen-like *hgcA* genes were assigned that function based on the consistency of that phylogenetic group (Gilmour et al., 2018; McDaniel et al., 2020). These classifications should be interpreted with caution, because in addition to the possibility of HGT, the metabolic potential of organisms can vary widely within phylogenetically similar groups based on differential gene content.

Statistical analyses

Linear regression of MeHg to *hgcA* abundance was performed on log-log transformed data using the "lm" function in R (v4.1.3). We reported the adjusted R^2 value, which accounts for the number of variables relative to the number of observations. Inspection of the QQplot for the residuals of the models indicates a slight left skew, likely driven by the higher proportion of samples collected from the oxic regions where MeHg and *hgcA* both near the detection limit and by the outliers well below the curve where factors other than *hgcA* abundance are likely to strongly influence MeHg concentrations.

Supplementary Results and Discussion

Fermentative hgcA+ mOTUs

Five of the hgcA+ mOTUs corresponding to putative fermentative organisms fell within the Verrucomicrobia phylum (Fig. 5; Table S6). The bins within these mOTUs were highly complete, all over 70% with at least one bin within each mOTU over 84%, with low redundancy (all under 10%, most under 3%). Additionally, the HgcA amino acid phylogeny is consistent with the bin phylogeny (Fig. S17). Collectively, this provides a high degree of confidence in the presence of *hgcA* in these organisms.

Kiritimatiellae: Four of the hgcA+ mOTUs were in the Kiritimatiellae class. Two, represented by HCC_KIRITI_008 and HCC_KIRITI_009, were recovered from the 2017 metagenomes and two, represented by HCC_KIRITI_010 and HCC_KIRITI_011, from the 2018 metagenomes (Fig. 5). The mOTUs from the two separate years are phylogenetically distinct (Fig. 5). These four bins all correspond to obligately fermentative organisms. They are all relatively complete (75 to 95% complete), making it unlikely that the absence of respiratory machinery in the 8 bins that make up these four OTUs is due to incompletely assembly and binning. There are some distinct metabolic differences between the two groups from the two different years, however. The two 2017 Kiritimatiellae mOTUs had the terminal oxidase *cydAB* and 8-11 multiheme cytochrome c genes. This suggests that these organisms may be tolerant of oxygen or other oxidants since they do not appear to have the capacity for respiration. Under high flow conditions such as 2017, increased turbulent mixing may cause entrainment of oxygen in the deeper waters, presenting an oxidative stressor. One of the mOTUs, HCC_KIRITI_008, also contained *nrfHA*. Interestingly, the main difference in abundance between the two mOTUs from 2017 is that HCC_KIRITI_008 showed a larger peak in the metalimnion at RM286 (Fig. 5).

This could indicate a greater tolerance of higher redox conditions, potentially due to the ability to detoxify nitrite. On the other hand, the two hgcA+ Kiritimatiellae from 2018 carried no terminal oxidases and had no multi-heme cytochrome c genes. While they are still present under nitrate-reducing conditions, the lower flow conditions in 2018 may have reduced the oxidative stresses they encountered in the metalimnion. The hgcA+ Kiritimatiellae appear to have been polysaccharide-degrading organisms, as each mOTU had at least 85 glycoside hydrolases (GHs) apart from HCC_KIRITI_010, which had 31. Each of the Kiritimatiellae clustered phylogenetically with highly abundant hgcA+ mOTUs from the anoxic hypolimnion of a eutrophic freshwater lake (Peterson et al., 2020). Two more distantly related Kiritimatiellae were also identified in another eutrophic freshwater lake (Jones et al., 2019), suggesting that hgcA+ organisms from this class may be a common member of the *hgcA*-carrying microbial community in freshwater systems, particularly in eutrophic systems with anoxic hypolimnia. We also recovered 8 Kiritimatiellae-associated mOTUs that did not carry *hgcA* (hgcA-). The hgcA- Kiritimatiellae showed a comparable phylogeny to the hgcA+ Kiritimatiellae, similar abundance profiles in Brownlee, and had similar metabolic capabilities (Fig. 5). This scattershot distribution of *hgcA* is consistent with previous work (McDaniel et al., 2020; Podar et al., 2015). The gene neighborhood around the *hgcA* sequences within the Kiritimatiellae mOTUs included *arsR*-like transcriptional regulators, arsenite transporters, arsenite reductase, genes thought to be involved in arsenic transformation such as cytochrome c biogenesis-like proteins and certain thioredoxins, and metal efflux pumps (Fig. S17). Overall, this synteny is consistent with that observed in other hgcA+ organisms elsewhere (Gionfriddo, Stott, et al., 2020; Goñi-Urriza et al., 2020; McDaniel et al., 2020).

Lentisphaeria: The fifth mOTU in the Verrucomicrobia phylum was HCC_LENTI_007, which was classified into the Lentisphaeria class. This mOTU corresponds to an obligately fermentative organism, as it had no electron transport chain, but appeared well-adapted to high-redox conditions; it was most abundant at 45 m at RM286 in 2017, where nitrate levels were still around 0.6 mg_N/L (Fig. 5) and encoded an anaerobic sulfite reductase and the *cydAB* terminal oxidase. We also recovered an hgcA- Lentisphaeria; however, it was phylogenetically distinct and exhibited a much different abundance pattern. Unlike the Kiritimatiellae mOTUs, HCC_LENTI_007 did not appear to specialize in polysaccharide degradation with only 22 GHs. The closest reference genome to HCC_LENTI_007 was an hgcA+ Lentisphaeria from the same eutrophic freshwater lake where the Kiritimatiellae were recovered (Peterson et al., 2020). This suggests that Lentisphaeria are a ubiquitous member of the *hgcA*-carrying microbial community in eutrophic freshwater systems, although not at the same abundance as Kiritimatiellae.

High-redox respiratory hgcA+ mOTUs

Prolixibacteraceae: The HCC_PROLIX_006 mOTU was recovered in 2018 and was classified into the Prolixibacteraceae family within the Bacteroidales order (NCBI taxonomy places Prolixibacteraceae within Marinilabiliales) (Fig. 6a). Six additional closely related mOTUs were recovered that did not contain the *hgcA* gene (Fig. 6a). To examine the distribution of hgcA within this family, we retrieved 134 reference genomes and mOTUs from NCBI that are classified within the Prolixibacteraceae order. GTDB classified 91 of these as Prolixibacteraceae (which is consistent with the mOTU phylogeny). Of these, only six contained *hgcA*, with an additional hgcA+ mOTU from the 43 mOTUs annotated as Prolixibacteraceae by NCBI (Fig. S18). These seven sequences clustered together with strong bootstrap support distinct from other

Bacteroidales-associated HgcA sequences, except for a Dysgonomonadaceae- and a Paludibacter-associated HgcA sequence (Fig. 6b). All these *hgcAB* gene pairs were located between an arsenate reductase and an arsenite transporter, and some were preceded with an *arsR*-like transcriptional regulator. This association between *hgcAB* and arsenic cycling genes has been previously noted (Gionfriddo, Stott, et al., 2020; Goñi-Urriza et al., 2020; McDaniel et al., 2020) and suggests a possible link between the biogeochemical cycling of these two elements. HCC_PROLIX_006 was most abundant in the metalimnion at RM286 in 2018, where MeHg was at a local maximum (0.315 ng/L) and nitrate was at a local minimum, but still 0.56 mg_N/L (Fig. 6c). It also had a small peak in abundance at RM300 in the metalimnion, just where nitrate dropped below detection and MeHg peaked (Fig. 6c). This abundance pattern suggests that the corresponding organism is suited to nitrate-reducing conditions and a potential driver for the elevated MeHg levels at this location.

Indeed, the mOTU genome included membrane-bound nitrate reductase (*narGHI*) and a full electron transport chain to support nitrate respiration (Fig. 6d). Interestingly, it did not have the remaining genes for denitrification, but rather a *narK*-like nitrate-nitrite antiporter, suggesting that this organism just reduced nitrate to nitrite, then expelled nitrite from the cell. It also had several genes that encode pathways for external electron transfer (EET), including an outer-membrane multiheme cytochrome c (MHC) genes annotated as *extA* (Jiménez Otero et al., 2018) with an adjacent periplasmic MHC and the *imcH* gene, which has been shown to be used for EET to high-redox extracellular terminal electron acceptors, such as Mn oxides (Levar et al., 2014). A complete gene cluster for a molybdopterin oxidoreductase, homologous to the tetrathiosulfate reductase *phs*, suggests the potential for other reductive terminal electron accepting processes, particularly those involving oxyanions. The genome also included *cbb_3* and

aa_3 cytochrome c oxidases; however, the organism's preference for oxygen-depleted conditions suggests these are used for oxygen detoxification. Additionally, the genome lacked pyruvate dehydrogenase, instead using a pyruvate:ferredoxin oxidoreductase (PFOR), which is associated with pyruvate metabolism under anaerobic conditions and is inactivated by oxygen (Vita et al., 2008). The genome also includes 248 GHs, suggesting a role as a primary degrader of large polymeric carbohydrates. The genome has a complete glycolytic pathway, a nearly complete TCA cycle, and pentose phosphate pathway, suggesting that the organism can completely oxidize the resulting monomers it generates using the GHs and is not dependent on fermentative organisms. We also identified the acetyl-CoA synthetase (ACS), 5,10-methylene-H4F reductase (Met), and 5,10-methenyl-H4F cyclohydrolase (FolD) genes, together which are capable of converting acetate into formyltetrahydrofolate, the purported methyl donor for MeHg formation; however, no formate dehydrogenase or 10-formyl-H4F synthetase genes were identified, which would complete the traditional Wood-Ljundahl pathway (Ragsdale, 2008).

Pelobacteraceae: HCC_PELOB_005 was reconstructed from 2019 metagenomes and was classified as Pelobacteraceae (Fig. S19). It was most abundant at 50m at RM300 in 2019, where filter-passing Mn was at a local maximum, suggesting a potential hotspot for Mn cycling. HCC_PELOB_005 contained two potential complexes that could mediate EET. The first one consisted of *extABCD* homologues, which encode a porin-cytochrome c complex (Jiménez Otero et al., 2018). HCC_PELOB_005 also carried a second gene cluster that included the following genes: molybdopterin oxidoreductase, iron-sulfur protein, and cytochrome b6, all cytoplasmic; a membrane-bound *nrfD*-like protein; two periplasmic cytochrome c genes, as well as an unknown periplasmic-localized gene; and a large extracellular cytochrome c. While the function of this

cluster is unknown, the membrane-spanning nature of it suggests it could mediate some form of external electron transfer. HCC_PELOB_005 also included *imcH* and *cbcL*, which facilitate EET to high and low redox TEAPs, respectively (Levar et al., 2017). Overall, it includes 23 multiheme cytochrome c (MHC) genes, which are often linked to EET processes (He et al., 2017). This bin also encodes a *coxACDB* terminal oxidase and *nrfAH*. While these could be used for respiration of oxygen or nitrite, respectively, both genes can be found in metal-reducing Geobacteraceae and are thought to be involved in oxygen/nitrite detoxification (Aklujkar et al., 2010). Like HCC_PROLIX_006, HCC_PELOB_005 contains a PFOR rather than pyruvate dehydrogenase, further suggesting it is an anaerobic organism.

Sulfate-reducing hgcA+ mOTUs

Desulfobacterales: Three hgcA+ mOTUs were classified in the Desulfobacterales order. The metabolic assessment of these was limited by the fact that these genomes were only 68-75% complete. Each of these bins had at least partial reductive dissimilatory sulfite reduction (*dsr*) operons and two, HCCC_DESULF_002 and HCCC_DESULF_004, had adenylyl-sulfate reductase (*aprA*), which is directly responsible for reducing sulfate. All three Desulfobacterales contained a molybdopterin oxidoreductases phylogenetically similar to thiosulfate reductase (*phs*). They also each included an electron transport chain, including either complex I or the *rnf* operon. Each mOTU also potential oxidant detoxification mechanisms such as *cydAB* or *nrfHA*. There were no Desulfobacterales identified that did not contain *hgcA*.

Smithellaceae: There was one hgcA+ mOTU, designated HCC_SYNTR_001, recovered from 2019 that was classified into the Smithellaceae family within the Syntrophales order. While the genome did contain *dsrABD*, suggesting it as a sulfate-reducing organism, it did not contain

apr, *sat*, or an electron transport chain, despite being predicted to be 91% complete. Combined with the fact that this organism was detected at 56 m at RM300, despite a lack of sulfide accumulation and about 1.4 mgN/L of nitrate present, this suggests that this organism may not be a sulfate-reducing despite the *dsrABD* genes, which is the standard marker used for sulfate reduction.

References

Aklujkar, M., Young, N. D., Holmes, D., Chavan, M., Risso, C., Kiss, H. E., Han, C. S., Land, M. L., & Lovley, D. R. (2010). The genome of *Geobacter bemidjiensis*, exemplar for the subsurface clade of *Geobacter* species that predominate in Fe(III)-reducing subsurface environments. *BMC Genomics*, *11*(1), 490. https://doi.org/10.1186/1471-2164-11-490

Anantharaman, K., Brown, C. T., Hug, L. A., Sharon, I., Castelle, C. J., Probst, A. J., Thomas, B. C., Singh, A., Wilkins, M. J., Karaoz, U., Brodie, E. L., Williams, K. H., Hubbard, S. S., & Banfield, J. F. (2016). Thousands of microbial genomes shed light on interconnected biogeochemical processes in an aquifer system. *Nature Communications*, *7*(13219), 1–11. https://doi.org/10.1038/ncomms13219

Aramaki, T., Blanc-Mathieu, R., Endo, H., Ohkubo, K., Kanehisa, M., Goto, S., & Ogata, H. (2020). KofamKOALA: KEGG Ortholog assignment based on profile HMM and adaptive score threshold. *Bioinformatics*, *36*(7), 2251–2252. https://doi.org/10.1093/bioinformatics/btz859

Bowers, R. M., Stepanauskas, R., Harmon-Smith, M., Doud, D., Reddy, T. B. K., Schulz, F., Jarett, J., Rivers, A. R., Eloe-Fadrosh, E. A., Tringe, S. G., Ivanova, N. N., Copeland, A., Clum, A., Becraft, E. D., Malmstrom, R. R., Birren, B., Podar, M., Bork, P., Weinstock, G. M., … Woyke, T. (2017). Minimum information about a single amplified genome (MISAG) and a metagenome-assembled genome (MIMAG) of bacteria and archaea. *Nature Biotechnology*, *35*(8), 725–731. https://doi.org/10.1038/nbt.3893

Chaumeil, P.-A., Mussig, A. J., Hugenholtz, P., & Parks, D. H. (2019). GTDB-Tk: A toolkit to classify genomes with the Genome Taxonomy Database. *Bioinformatics*, *36*(6), 1925–1927. https://doi.org/10.1093/bioinformatics/btz848

Chen, S., Zhou, Y., Chen, Y., & Gu, J. (2018). fastp: An ultra-fast all-in-one FASTQ preprocessor. *Bioinformatics*, *34*(17), i884–i890. https://doi.org/10.1093/bioinformatics/bty560

Delmont, T. O., Quince, C., Shaiber, A., Esen, Ö. C., Lee, S. T., Rappé, M. S., McLellan, S. L., Lücker, S., & Eren, A. M. (2018). Nitrogen-fixing populations of Planctomycetes and Proteobacteria are abundant in surface ocean metagenomes. *Nature Microbiology*, *3*(7), 804–813. https://doi.org/10.1038/s41564-018-0176-9

DeWild, J. F., Olson, M. L., & Olund, S. D. (2002). *Determination of Methyl Mercury by Aqueous Phase Ethylation, Followed by Gas Chromatographic Separation with Cold Vapor Atomic Fluorescence Detection. Open-file Report* (No. 01–445; Open-File Report). U. S. Geological Survey.

Eddy, S. R. (2015). *Hmmer*. http://hmmer.org/

Edgar, R. C. (2004). MUSCLE: a multiple sequence alignment method with reduced time and space complexity. *BMC Bioinformatics*, *5*, 113. https://doi.org/10.1186/1471-2105-5-113

Eren, A. M., Esen, Ö. C., Quince, C., Vineis, J. H., Morrison, H. G., Sogin, M. L., & Delmont, T. O. (2015). Anvi'o: An advanced analysis and visualization platform for 'omics data. *PeerJ*, *3*, e1319. https://doi.org/10.7717/peerj.1319

Garbarino, J. R., & Hoffman, G. L. (1999). *Methods of analysis by the U.S. Geological Survey National Water Quality Laboratory -comparison of a nitric acid in-bottle digestion procedure to other whole-water digestion procedures* (Open-File Report No. 99–094; Open-File Report). U.S. Geological Survey.

Gilmour, C. C., Bullock, A. L., McBurney, A., Podar, M., & Elias, D. A. (2018). Robust mercury methylation across diverse methanogenic *Archaea*. *MBio*, *9*(2), e02403-17. https://doi.org/10.1128/mBio.02403-17

Gionfriddo, C. M., Capo, E., Peterson, B. D., Heyu, L., Jones, D. S., Bravo, A. G., Bertilsson, S., Moreau, J. W., McMahon, K. D., Elias, D. A., & Gilmour, C. C. (2021). *Hg-MATE-Db.v1.01142021*. https://doi.org/10.25573/serc.13105370.v1

Gionfriddo, C. M., Stott, M. B., Power, J. F., Ogorek, J. M., Krabbenhoft, D. P., Wick, R., Holt, K., Chen, L.-X., Thomas, B. C., Banfield, J. F., & Moreau, J. W. (2020). Genome-resolved metagenomics and detailed geochemical speciation analyses yield new insights into microbial mercury cycling in geothermal springs. *Applied and Environmental Microbiology*, *86*(15), e00176-20. https://doi.org/10.1128/AEM.00176-20

Gionfriddo, C. M., Wymore, A. M., Jones, D. S., Wilpiszeski, R. L., Lynes, M. M., Christensen, G. A., Soren, A., Gilmour, C. C., Podar, M., & Elias, D. A. (2020). An improved *hgcAB* primer set and direct high-throughput sequencing expand Hg-methylator diversity in nature. *Frontiers in Microbiology*, *11*, 541554. https://doi.org/10.3389/fmicb.2020.541554

Goñi-Urriza, M., Klopp, C., Ranchou-Peyruse, M., Ranchou-Peyruse, A., Monperrus, M., Khalfaoui-Hassani, B., & Guyoneaud, R. (2020). Genome insights of mercury methylation among *Desulfovibrio* and *Pseudodesulfovibrio* strains. *Research in Microbiology*, *171*(1), 3–12. https://doi.org/10.1016/j.resmic.2019.10.003

He, S., Stevens, S. L. R., Chan, L.-K., & Bertilsson, S. (2017). Ecophysiology of freshwater Verrucomicrobia inferred from metagenome-assembled genomes. *MSphere*, *2*(5), 1–17. https://doi.org/10.1128/mSphere.00277-17

Horvat, M., Bloom, N. S., & Liang, L. (1993). Comparison of distillation with other current isolation methods for the determination of methyl mercury compounds in low level environmental samples. *Analytica Chimica Acta*, *281*, 135–152. https://doi.org/10.1016/0003-2670(93)85348-N

Hyatt, D., Chen, G.-L., LoCascio, P. F., Land, M. L., Larimer, F. W., & Hauser, L. J. (2010). Prodigal: Prokaryotic gene recognition and translation initiation site identification. *BMC Bioinformatics*, *11*, 119. https://doi.org/10.1186/1471-2105-11-119

Jiménez Otero, F., Chan, C. H., & Bond, D. R. (2018). Identification of different putative outer membrane electron conduits necessary for Fe(III) citrate, Fe(III) oxide, Mn(IV) oxide, or electrode reduction by *Geobacter sulfurreducens*. *Journal of Bacteriology*, *200*(19), e00347-18. https://doi.org/10.1128/JB.00347-18

Jones, D. S., Walker, G. M., Johnson, N. W., Mitchell, C. P. J., Coleman Wasik, J. K., & Bailey, J. V. (2019). Molecular evidence for novel mercury methylating microorganisms in sulfate-impacted lakes. *The ISME Journal*, *13*, 1659–1675. https://doi.org/10.1038/s41396-019-0376-1

Kang, D. D., Li, F., Kirton, E., Thomas, A., Egan, R., An, H., & Wang, Z. (2019). MetaBAT 2: An adaptive binning algorithm for robust and efficient genome reconstruction from metagenome assemblies. *PeerJ*, *7*, e7359. https://doi.org/10.7717/peerj.7359

Langmead, B., & Salzberg, S. L. (2012). Fast gapped-read alignment with Bowtie 2. *Nature Methods*, *9*(4), 357–359. https://doi.org/10.1038/nmeth.1923

Lepak, R. F., Krabbenhoft, D. P., Ogorek, J. M., Tate, M. T., Bootsma, H. A., & Hurley, J. P. (2015). Influence of *Cladophora*–quagga mussel assemblages on nearshore

methylmercury production in Lake Michigan. *Environmental Science & Technology*, *49*(13), 7606–7613. https://doi.org/10.1021/es506253v

Levar, C. E., Chan, C. H., Mehta-Kolte, M. G., & Bond, D. R. (2014). An inner membrane cytochrome required only for reduction of high redox potential extracellular electron acceptors. *MBio*, *5*(6). https://doi.org/10.1128/mBio.02034-14

Levar, C. E., Hoffman, C. L., Dunshee, A. J., Toner, B. M., & Bond, D. R. (2017). Redox potential as a master variable controlling pathways of metal reduction by Geobacter sulfurreducens. *The ISME Journal*, *11*(3), 741–752. https://doi.org/10.1038/ismej.2016.146

Lever, M. A., Torti, A., Eickenbusch, P., Michaud, A. B., Šantl-Temkiv, T., & Jørgensen, B. B. (2015). A modular method for the extraction of DNA and RNA, and the separation of DNA pools from diverse environmental sample types. *Frontiers in Microbiology*, *6*, 476. https://doi.org/10.3389/fmicb.2015.00476

Li, H., Handsaker, B., Wysoker, A., Fennell, T., Ruan, J., Homer, N., Marth, G., Abecasis, G., Durbin, R., & 1000 Genome Project Data Processing Subgroup. (2009). The Sequence Alignment/Map format and SAMtools. *Bioinformatics*, *25*(16), 2078–2079. https://doi.org/10.1093/bioinformatics/btp352

Matsen, F. A., Kodner, R. B., & Armbrust, E. V. (2010). pplacer: Linear time maximum-likelihood and Bayesian phylogenetic placement of sequences onto a fixed reference tree. *BMC Bioinformatics*, *11*(538), 1–16. https://doi.org/10.1186/1471-2105-11-538

McDaniel, E. A., Peterson, B. D., Stevens, S. L. R., Tran, P. Q., Anantharaman, K., & McMahon, K. D. (2020). Expanded phylogenetic diversity and metabolic flexibility of

mercury-methylating microorganisms. *MSystems*, *5*(4), e00299-20. https://doi.org/10.1128/mSystems.00299-20

Müller, A. L., Kjeldsen, K. U., Rattei, T., Pester, M., & Loy, A. (2015). Phylogenetic and environmental diversity of DsrAB-type dissimilatory (bi)sulfite reductases. *The ISME Journal*, *9*(5), 1152–1165. https://doi.org/10.1038/ismej.2014.208

Naymik, J., Larsen, C. A., Myers, R., Hoovestol, C., Gastelecutto, N., & Bates, D. (2023). Long-term trends in inflowing chlorophyll *a* and nutrients and their relation to dissolved oxygen in a large western reservoir. *Lake and Reservoir Management*, *39*(1), 53–71. https://doi.org/10.1080/10402381.2022.2160395

Nurk, S., Meleshko, D., Korobeynikov, A., & Pevzner, P. A. (2017). metaSPAdes: A new versatile metagenomic assembler. *Genome Research*, *27*(5), 824–834. https://doi.org/10.1101/gr.213959.116

Olmsted, C. N., Ort, R., Tran, P. Q., McDaniel, E. A., Roden, E. E., Bond, D. R., He, S., & McMahon, K. D. (2022). Environmental predictors of electroactive bacterioplankton in small boreal lakes. *Environmental Microbiology*, *25*, 705–720. https://doi.org/10.1111/1462-2920.16314

Olund, S. D., DeWild, J. F., Olson, M. L., & Tate, M. T. (2004). Methods for the preparation and analysis of solids and suspended solids for total mercury. In *U.S. Geological Survey Techniques of Water-Resources Investigations, Book 5, Chapter A8*. U.S. Geological Survey.

Ondov, B. D., Treangen, T. J., Melsted, P., Mallonee, A. B., Bergman, N. H., Koren, S., & Phillippy, A. M. (2016). Mash: Fast genome and metagenome distance estimation using MinHash. *Genome Biology*, *17*, 132. https://doi.org/10.1186/s13059-016-0997-x

Parks, D. H., Chuvochina, M., Rinke, C., Mussig, A. J., Chaumeil, P.-A., & Hugenholtz, P. (2022). GTDB: An ongoing census of bacterial and archaeal diversity through a phylogenetically consistent, rank normalized and complete genome-based taxonomy. *Nucleic Acids Research*, *50*(Database issue), D785–D794. https://doi.org/10.1093/nar/gkab776

Parks, J. M., Johs, A., Podar, M., Bridou, R., Hurt, R. A., Smith, S. D., Tomanicek, S. J., Qian, Y., Brown, S. D., Brandt, C. C., Palumbo, A. V., Smith, J. C., Wall, J. D., Elias, D. A., & Liang, L. (2013). The genetic basis for bacterial mercury methylation. *Science*, *339*(6125), 1332–1335. https://doi.org/10.1126/science.1230667

Peterson, B. D., McDaniel, E. A., Schmidt, A. G., Lepak, R. F., Janssen, S. E., Tran, P. Q., Marick, R. A., Ogorek, J. M., DeWild, J. F., Krabbenhoft, D. P., & McMahon, K. D. (2020). Mercury methylation genes identified across diverse anaerobic microbial guilds in a eutrophic sulfate-enriched lake. *Environmental Science & Technology*, *54*, 15840–15851. https://doi.org/10.1021/acs.est.0c05435

Podar, M., Gilmour, C. C., Brandt, C. C., Soren, A., Brown, S. D., Crable, B. R., Palumbo, A. V., Somenahally, A. C., & Elias, D. A. (2015). Global prevalence and distribution of genes and microorganisms involved in mercury methylation. *Science Advances*, *1*, e1500675. https://doi.org/10.1126/sciadv.1500675

Price, M. N., Dehal, P. S., & Arkin, A. P. (2010). FastTree 2 – approximately maximum-likelihood trees for large alignments. *PLoS ONE*, *5*(3), e9490. https://doi.org/10.1371/journal.pone.0009490

Ragsdale, S. W. (2008). Enzymology of the Wood-Ljungdahl pathway of acetogenesis. *Annals of the New York Academy of Sciences*, *1125*(1), 129–136. https://doi.org/10.1196/annals.1419.015

Ranchou-Peyruse, M., Monperrus, M., Bridou, R., Duran, R., Amouroux, D., Salvado, J. C., & Guyoneaud, R. (2009). Overview of mercury methylation capacities among anaerobic bacteria including representatives of the sulphate-reducers: Implications for environmental studies. *Geomicrobiology Journal*, *26*(1), 1–8. https://doi.org/10.1080/01490450802599227

Sayers, E. W., Beck, J., Bolton, E. E., Bourexis, D., Brister, J. R., Canese, K., Comeau, D. C., Funk, K., Kim, S., Klimke, W., Marchler-Bauer, A., Landrum, M., Lathrop, S., Lu, Z., Madden, T. L., O'Leary, N., Phan, L., Rangwala, S. H., Schneider, V. A., … Sherry, S. T. (2021). Database resources of the National Center for Biotechnology Information. *Nucleic Acids Research*, *49*(Database issue), D10–D17. https://doi.org/10.1093/nar/gkaa892

Schliep, K. P. (2011). phangorn: Phylogenetic analysis in R. *Bioinformatics*, *27*(4), 592–593. https://doi.org/10.1093/bioinformatics/btq706

Sieber, C. M. K., Probst, A. J., Sharrar, A., Thomas, B. C., Hess, M., Tringe, S. G., & Banfield, J. F. (2018). Recovery of genomes from metagenomes via a dereplication, aggregation and scoring strategy. *Nature Microbiology*, *3*(7), 836–843. https://doi.org/10.1038/s41564-018-0171-1

Sorek, R., Zhu, Y., Creevey, C. J., Francino, M. P., Bork, P., & Rubin, E. M. (2007). Genome-wide experimental determination of barriers to horizontal gene transfer. *Science*, *318*(5855), 1449–1452. https://doi.org/10.1126/science.1147112

Stamatakis, A. (2014). RAxML version 8: A tool for phylogenetic analysis and post-analysis of large phylogenies. *Bioinformatics*, *30*(9), 1312–1313. https://doi.org/10.1093/bioinformatics/btu033

Thornton, K. W. (1990). Perspectives on reservoir limnology. In *Reservoir Limnology: Ecological Perspectives* (pp. 1–13). John Wiley & Sons.

U.S. EPA. (2002). *U.S. EPA Method 1631, Revision E: Mercury in Water by Oxidation, Purge and Trap, And Cold Vapor Atomic Fluorescence Spectrometry*. U.S. Environmental Protection Agency.

Varghese, N. J., Mukherjee, S., Ivanova, N., Konstantinidis, K. T., Mavrommatis, K., Kyrpides, N. C., & Pati, A. (2015). Microbial species delineation using whole genome sequences. *Nucleic Acids Research*, *43*(14), 6761–6771. https://doi.org/10.1093/nar/gkv657

Vita, N., Hatchikian, E. C., Nouailler, M., Dolla, A., & Pieulle, L. (2008). Disulfide bond-dependent mechanism of protection against oxidative stress in pyruvate-ferredoxin oxidoreductase of anaerobic *Desulfovibrio* bacteria. *Biochemistry*, *47*(3), 957–964. https://doi.org/10.1021/bi7014713

Weishaar, J. L., Aiken, G. R., Bergamaschi, B. A., Fram, M. S., Fujii, R., & Mopper, K. (2003). Evaluation of specific ultraviolet absorbance as an indicator of the chemical composition and reactivity of dissolved organic carbon. *Environmental Science & Technology*, *37*(20), 4702–4708. https://doi.org/10.1021/es030360x

Wu, Y.-W., Simmons, B. A., & Singer, S. W. (2016). MaxBin 2.0: An automated binning algorithm to recover genomes from multiple metagenomic datasets. *Bioinformatics*, *32*(4), 605–607. https://doi.org/10.1093/bioinformatics/btv638

Yu, G., Smith, D. K., Zhu, H., Guan, Y., & Lam, T. T. (2017). ggtree: An R package for visualization and annotation of phylogenetic trees with their covariates and other associated data. *Methods in Ecology and Evolution*, *8*, 28–36. https://doi.org/10.1111/2041-210X.12628

Zhou, Z., Tran, P. Q., Breister, A. M., Liu, Y., Kieft, K., Cowley, E. S., Karaoz, U., & Anantharaman, K. (2022). METABOLIC: High-throughput profiling of microbial genomes for functional traits, metabolism, biogeochemistry, and community-scale functional networks. *Microbiome*, *10*, 33. https://doi.org/10.1186/s40168-021-01213-8

Supplemental Tables

Table S1: Redox classification and geochemistry data. Includes all water chemistry measurements used in this manuscript. The full data set can be found in the U.S. Geological Survey data release [37]. Also includes the assigned redox status for each sample from this study.

Table S2: Metagenome metadata and read counts.

Table S3: Assembly metadata and statistics.

Table S4: Total coverage of metabolic genes of interest from all metagenomes.

Table S5: Characterization of all *hgcA* genes identified in assemblies.

Table S6: Taxonomy, abundance, completeness, metabolic gene content, and mOTU clustering data for all *hgcA*-containing bins.

Supplementary Figures

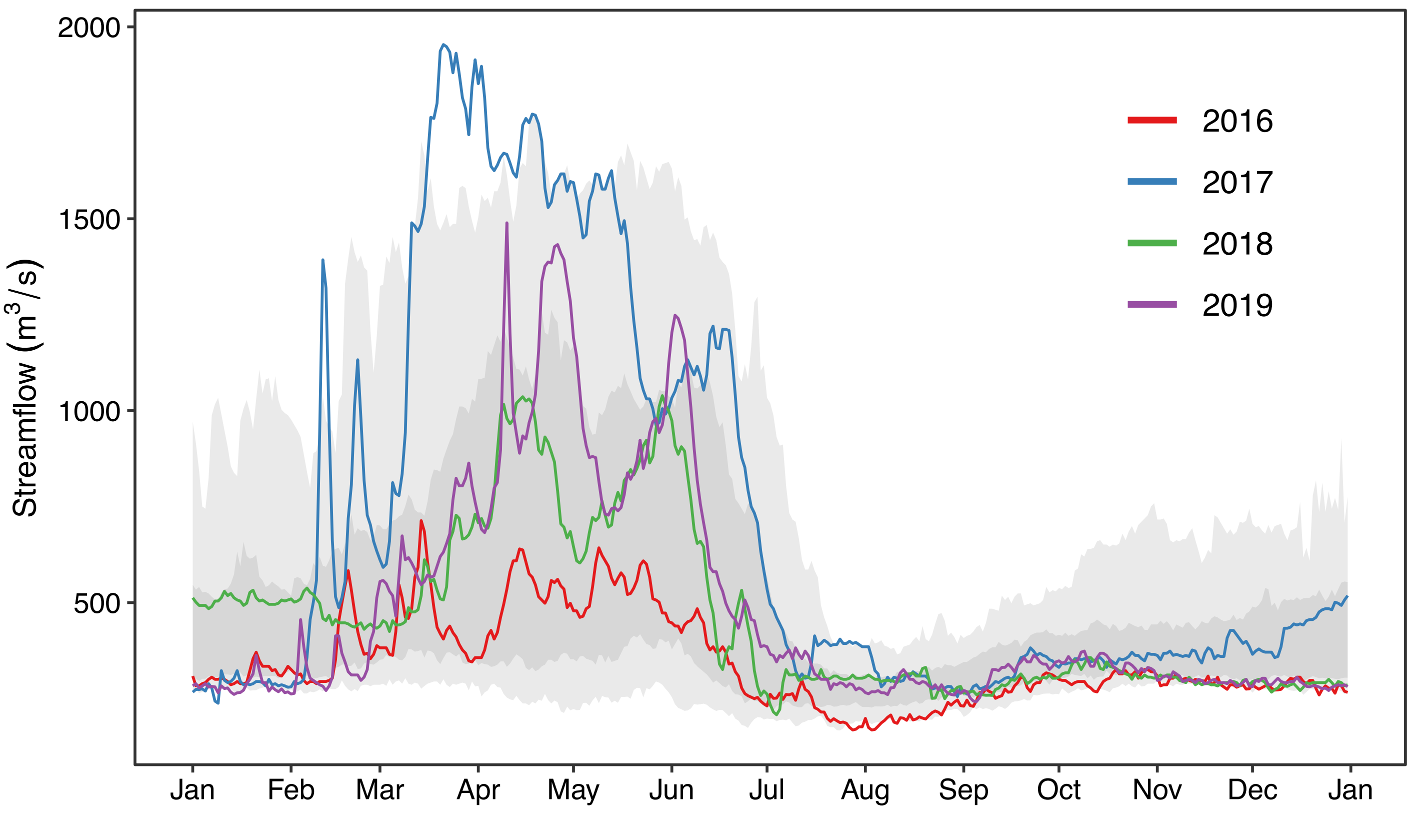

Figure S1. Historical streamflow and hydrographs of study years of the Snake River as measured at Weiser, Idaho. Average historical streamflows from 1968 to 2019 are shown in gray (50th percentile, dark gray; 90th percentile, light gray). Colored lines represent hydrograph of the Snake River over individual years included in this study.

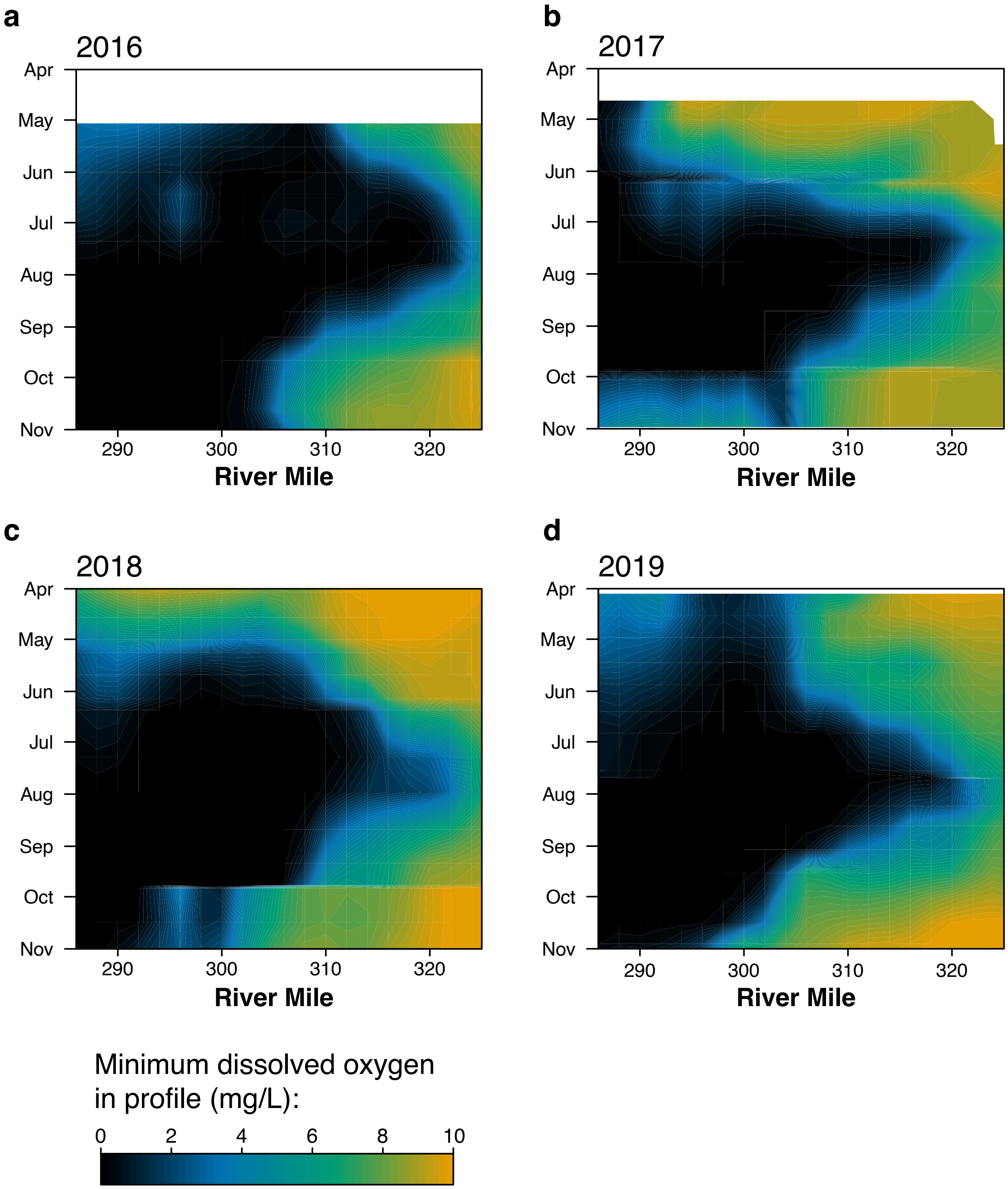

Figure S2. Onset of anoxia across Brownlee Reservoir for 2016 (**a**), 2017 (**b**), 2018 (**c**), and 2019 (**d**). Heatmap shows minimum dissolved oxygen (DO) concentration at any vertical point in the water column across the reservoir (x-axis) from April until the start of November (y-axis). Data is interpolated temporally and spatially based on continuous Seacat profiles collected every two river miles across Brownlee Reservoir every two weeks.

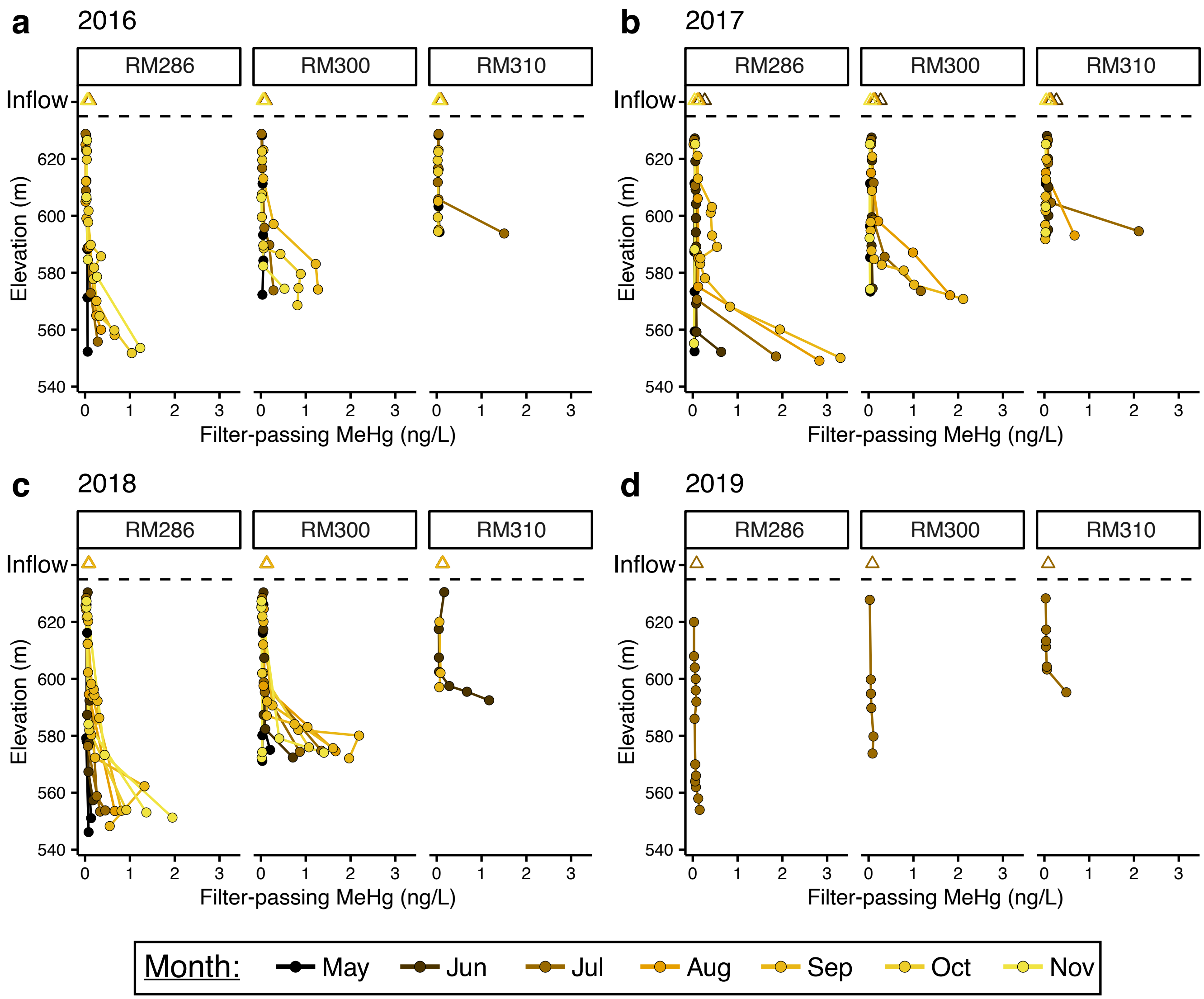

Figure S3. Progression of filter-passing MeHg accumulation in the water column across Brownlee Reservoir over the stratified period at RM286, RM300, and RM310 in 2016 (**a**), 2017 (**b**), 2018 (**c**), and 2019 (**d**). Open triangles show unfiltered MeHg concentrations at the inflow to the reservoir, which generally remain low throughout the year. Lines and points are colored by month collected, but some months may include multiple sampling trips. Lines connect data points collected on the same day.

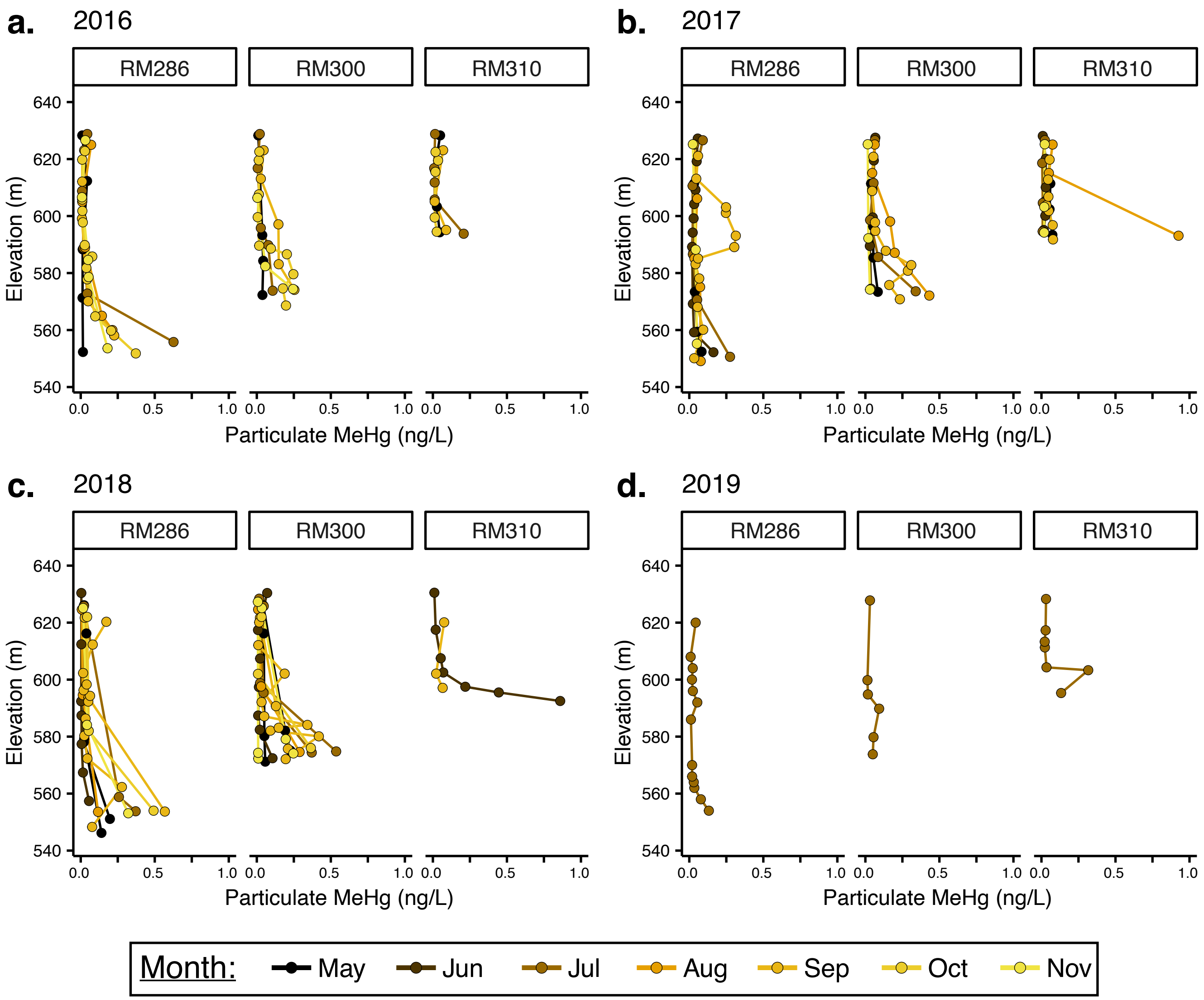

Figure S4. Particulate MeHg concentrations in the water column across Brownlee reservoir at RM286, RM300, and RM310 in 2016 (**a**), 2017 (**b**), 2018 (**c**), and 2019 (**d**). Lines and points are colored by the month of collection. Some months included multiple sampling events and therefore have multiple profiles. Lines connect data points collected on the same day.

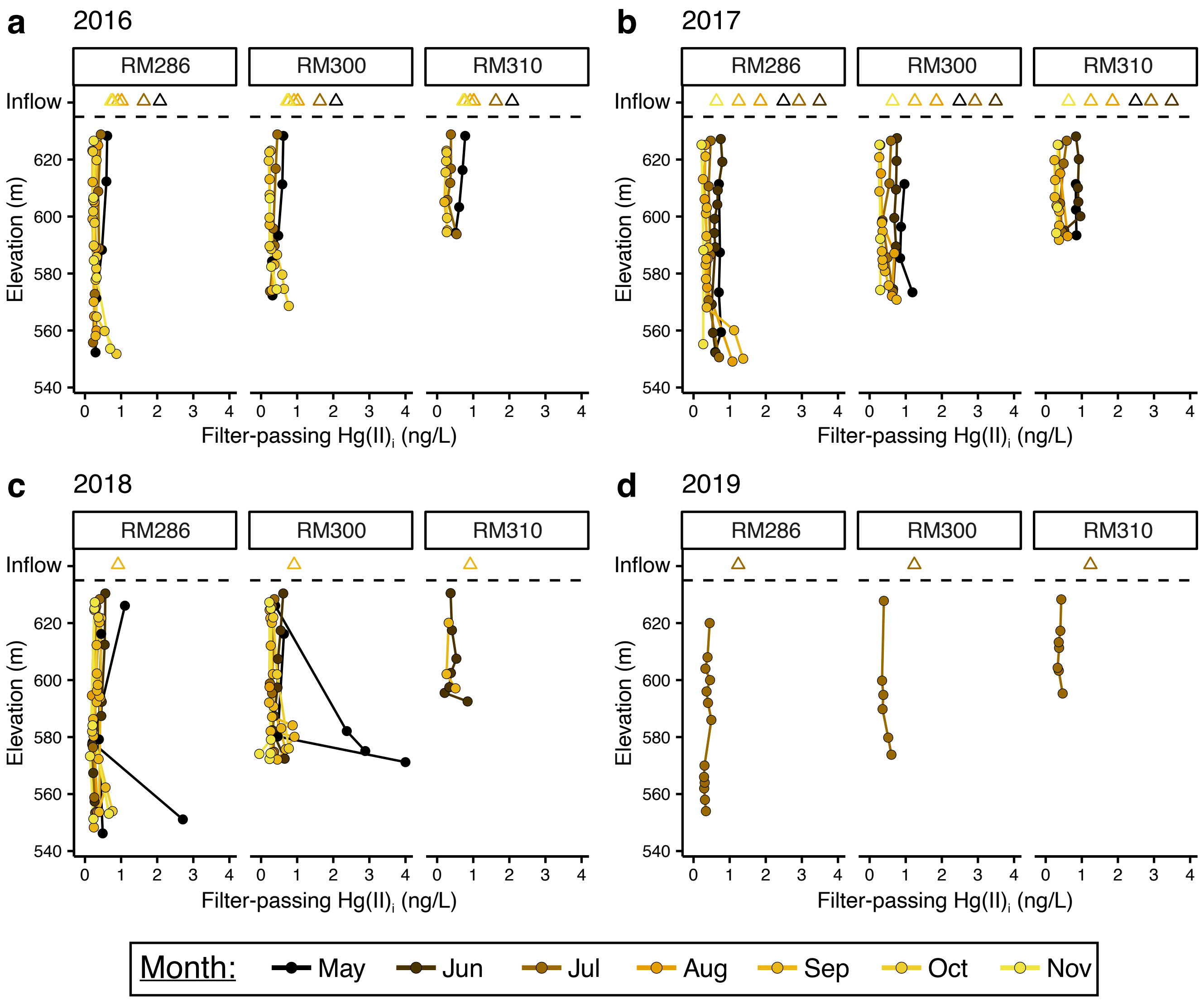

Figure S5. Progression of filter-passing Hg(II)$_i$ accumulation in the water column across Brownlee Reservoir over the stratified period at RM286, RM300, and RM310 in 2016 (**a**), 2017 (**b**), 2018 (**c**), and 2019 (**d**). Open triangles show unfiltered Hg(II)$_i$ concentrations at the reservoir inflow. Lines and points are colored by month collected, but some months may include multiple sampling trips. Lines connect data points collected on the same day.

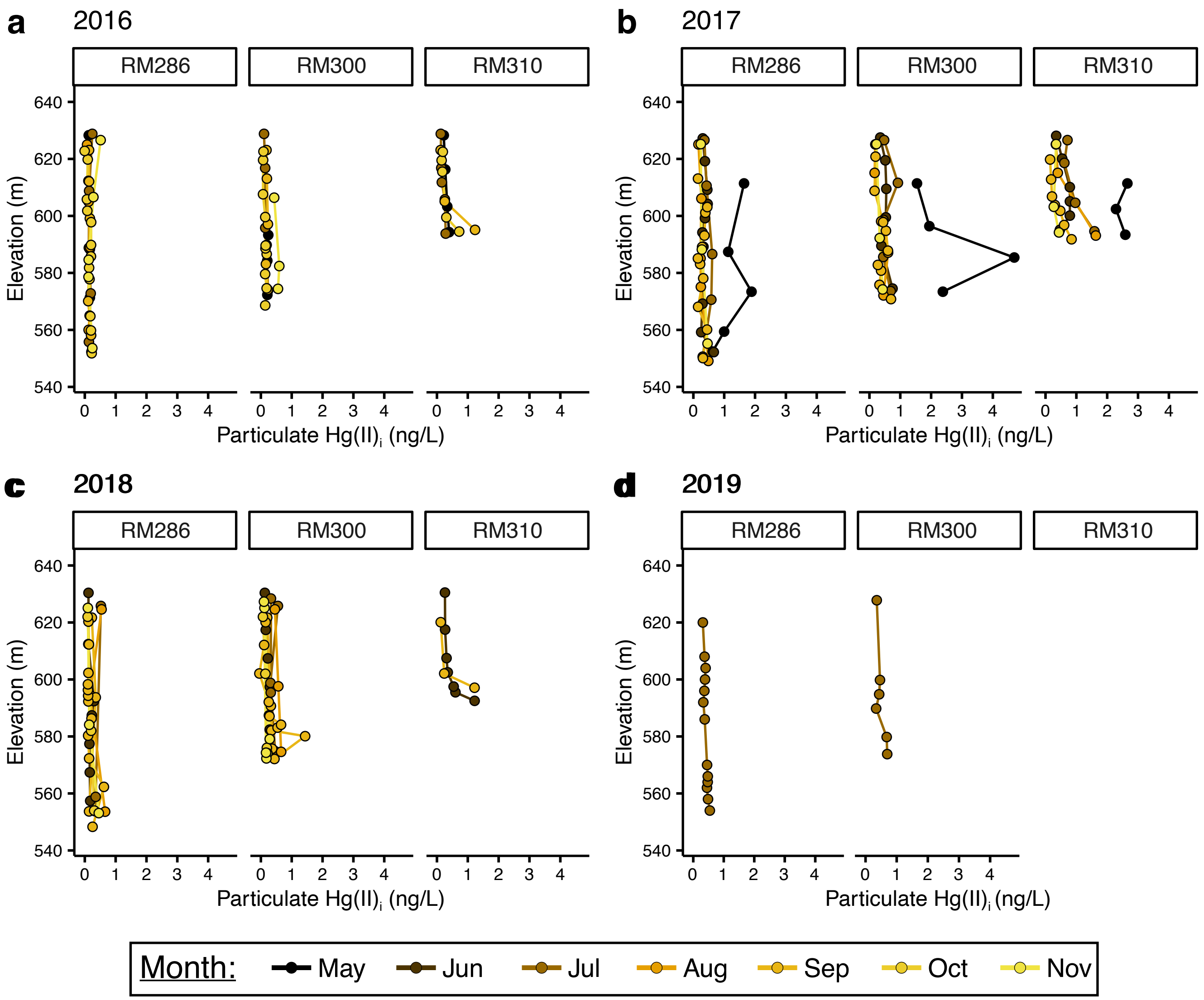

Figure S6. Progression of particulate inorganic Hg accumulation in the water column across Brownlee Reservoir over the stratified period at RM286, RM300, and RM310 in 2016 (**a**), 2017 (**b**), 2018 (**c**), and 2019 (**d**). Lines and points are colored by month collected, but some months may include multiple sampling trips. Lines connect data points collected on the same day.

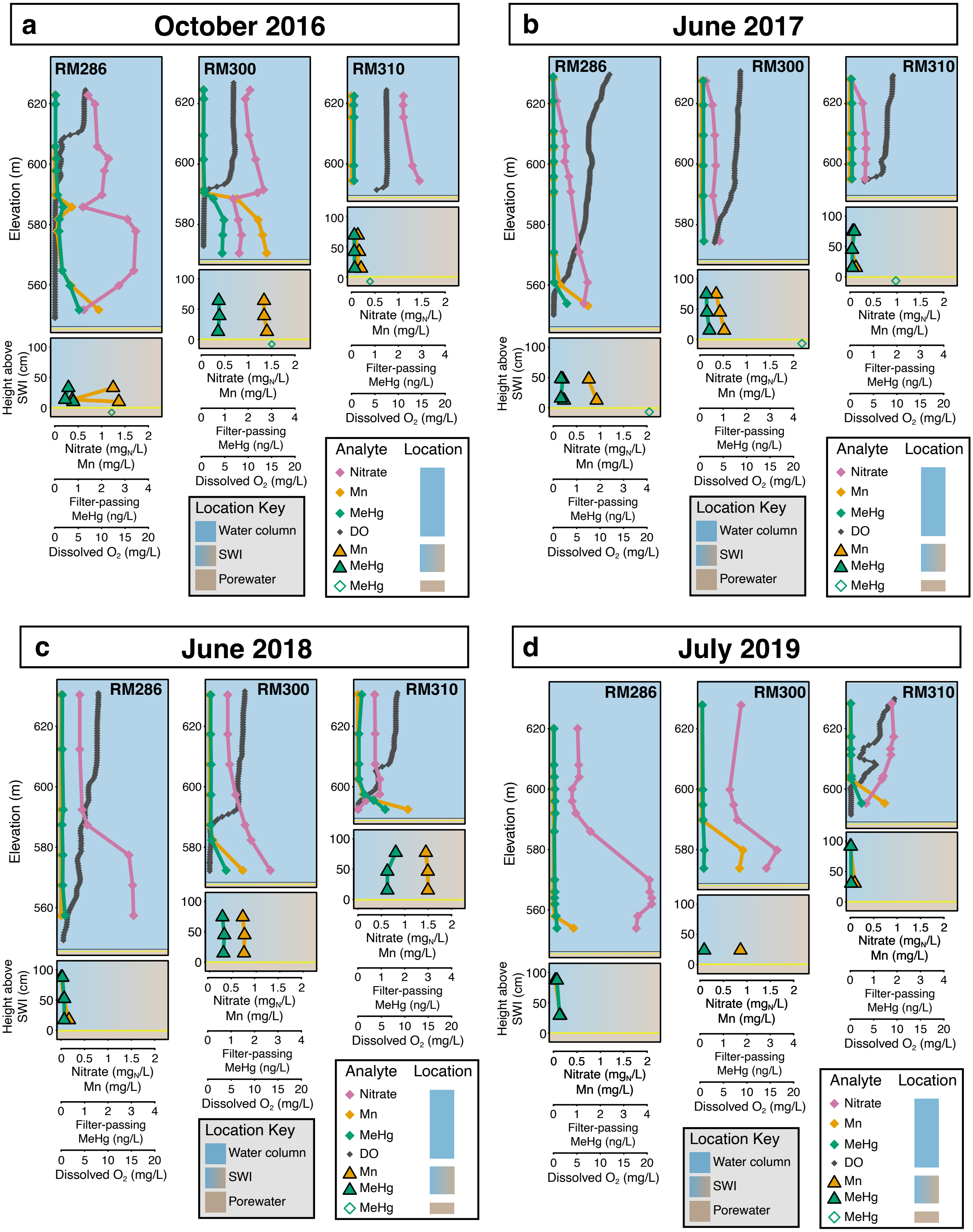

Figure S7. Additional redox profiles from Brownlee Reservoir during early stratification, in October 2016 (**a**), June 2017 (**b**), June 2018 (**c**), and July 2019 (**d**). Profiles shown were taken from summer or fall intensive sampling trips. Complete water chemistry data are available in the data release. Upper panels show water chemistry parameters in the water column. DO data was not available for RM286 and RM300 in July 2019. In the lower panels, the filled triangles represent water chemistry data from the water immediately overlying the sediment:water interface (SWI). The open diamonds show porewater concentrations, only available in 2016 and 2017. All Mn and MeHg values are for the filter-passing fraction. The yellow line shows the elevation of the sediment-water interface and the black line denotes the top of the SWI plot. The shading represents the areas designated as the water column, water overlying the SWI, or porewater.

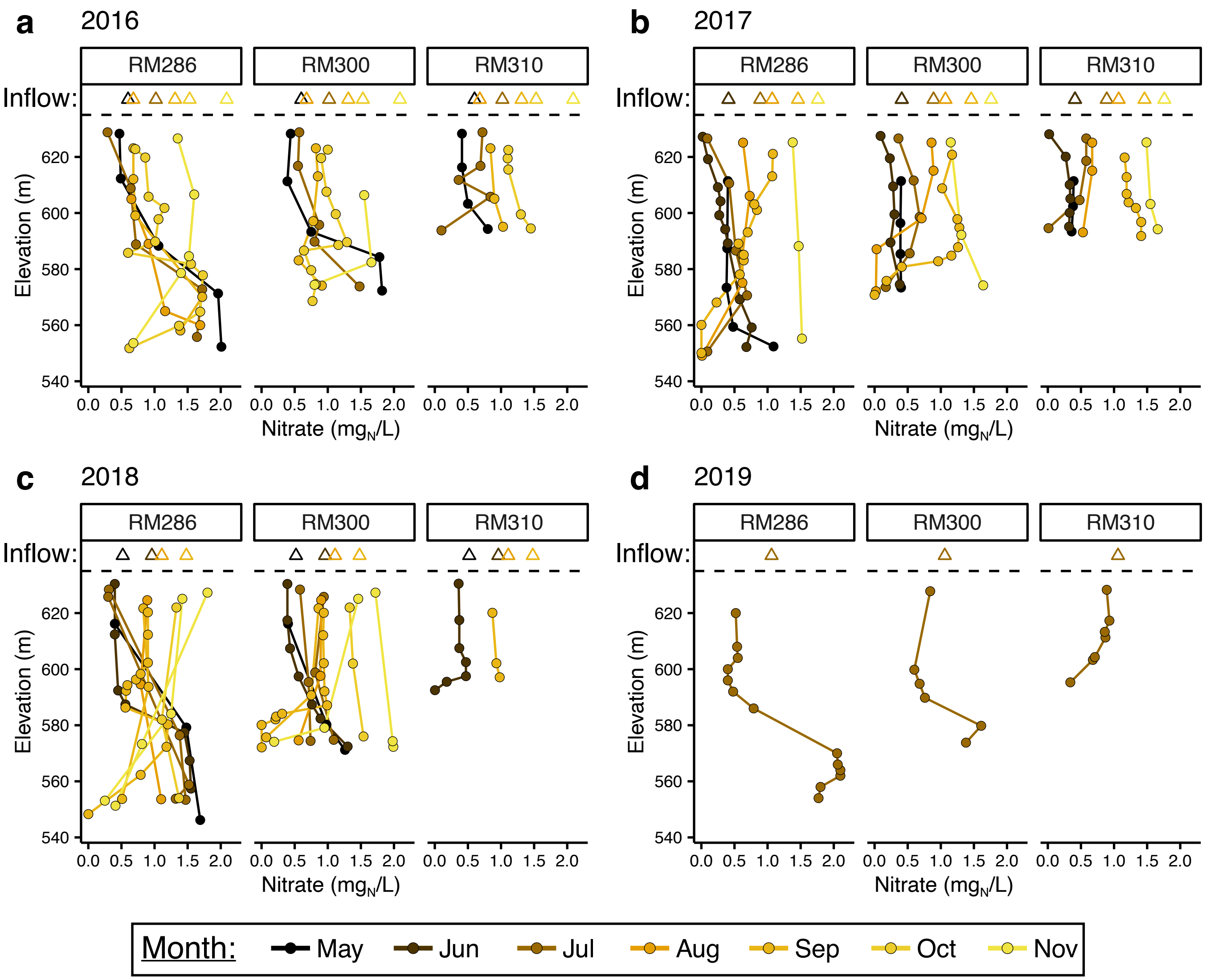

Figure S8. Nitrate concentrations in the water column across Brownlee Reservoir over each year at RM286, RM300, and RM310 in 2016 (**a**), 2017 (**b**), 2018 (**c**), and 2019 (**d**). Inflow concentrations of nitrate are shown above the profiles in the open triangles. Colors represent the month the profile was collected. Some months included more than one sampling event and thus have multiple profiles in the same color. Lines are shown between points from a single sampling event.

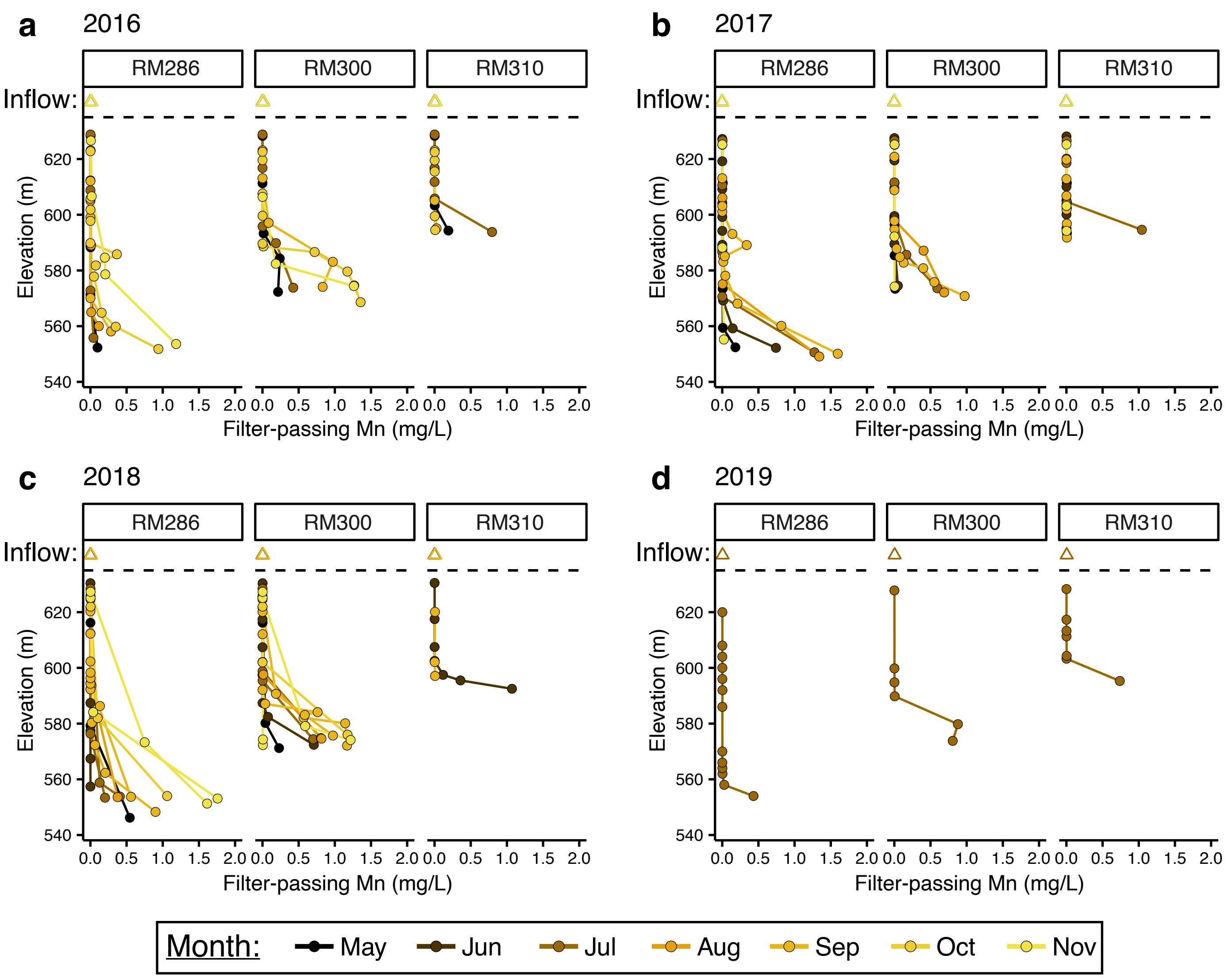

Figure S9. Filter-passing Mn concentrations at RM286, RM300, and RM310 in 2016 (**a**), 2017 (**b**), 2018 (**c**), and 2019 (**d**) in Brownlee Reservoir. Concentrations of filter-passing Mn in inflowing waters are represented by the open triangles above the dashed line. Colors correspond to the month of collection. Some months included more than one sampling event, leading to multiple profiles with the same color. Lines connect points for samples from a single sampling event.

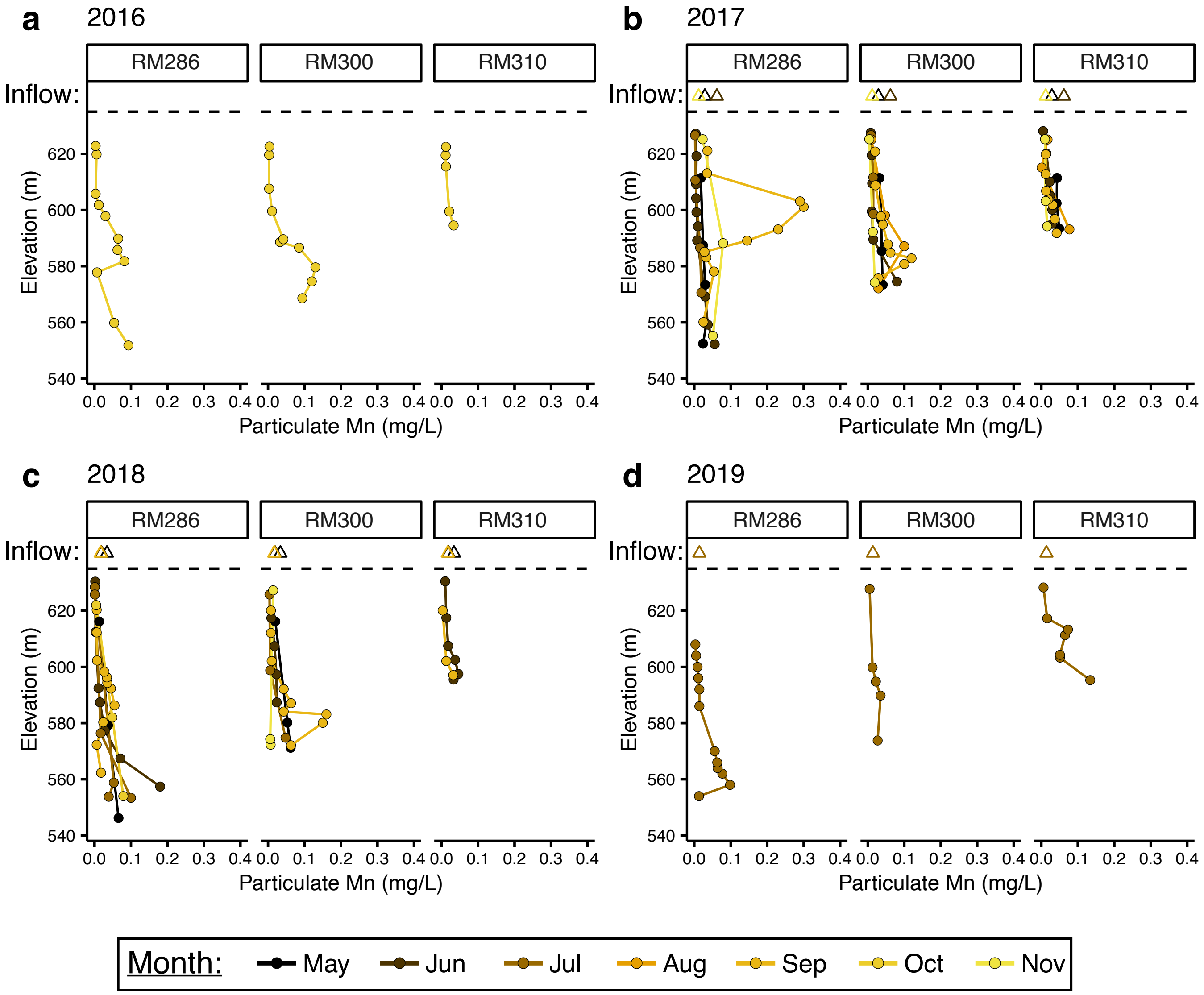

Figure S10. Particulate Mn concentrations at RM286, RM300, and RM310 in 2016 (**a**), 2017 (**b**), 2018 (**c**), and 2019 (**d**) in Brownlee Reservoir. Particulate Mn concentration at the inflow to the reservoir are shown above the profiles (open triangles). Colors represent the month of collection. On some occasions, multiple profiles were collected from a single month. Lines connect points for samples from a single sampling event.

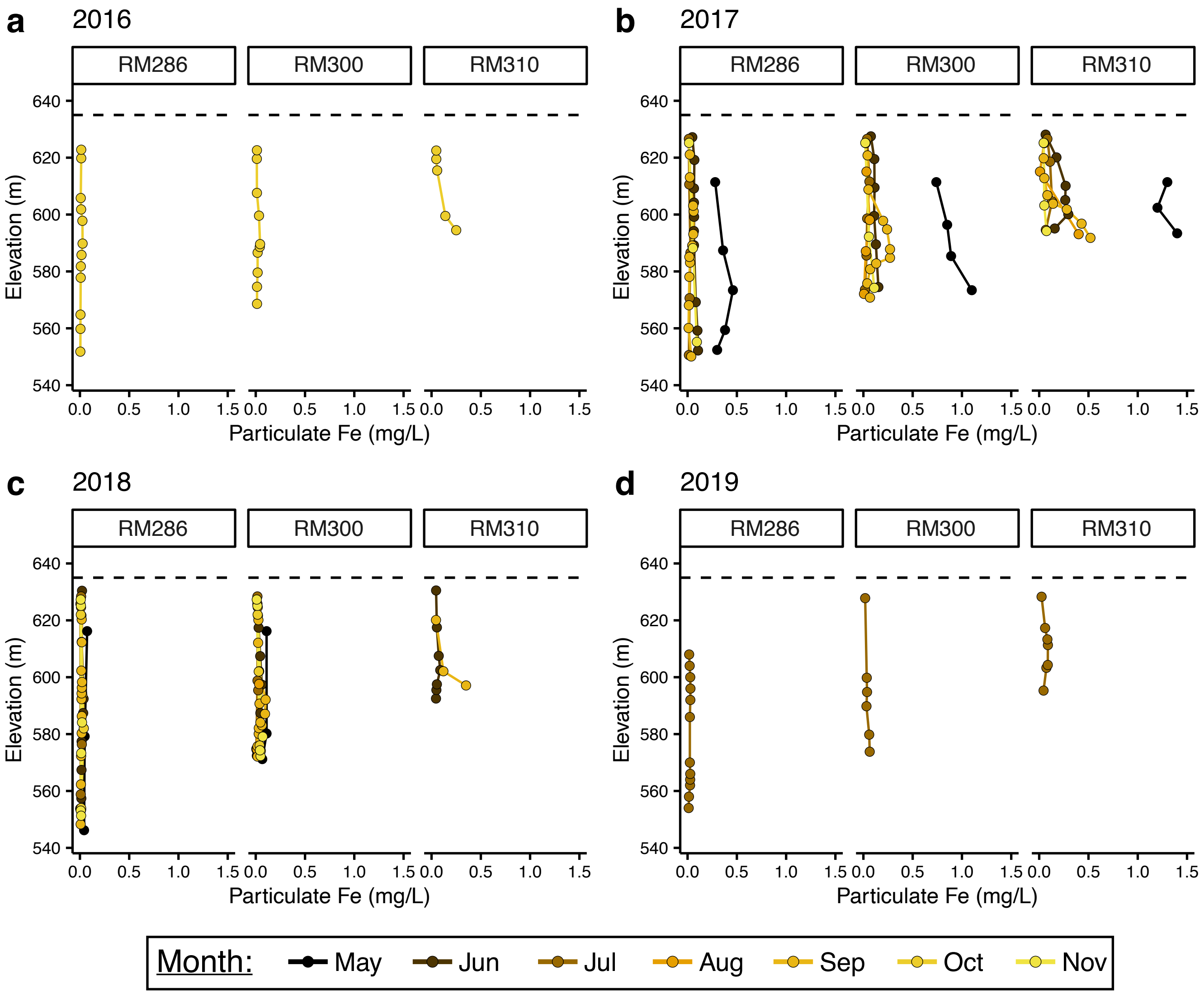

Figure S11. Particulate Fe concentrations at RM286, RM300, and RM310 in 2016 (**a**), 2017 (**b**), 2018 (**c**), and 2019 (**d**) in Brownlee Reservoir. Concentrations of particulate Fe in inflowing waters are presented above the dashed lines (open triangles). Collection month is represented by the color of the points. Sampling occurred more than one a month on occasion. Lines connect points for samples collected on the same sampling trip.

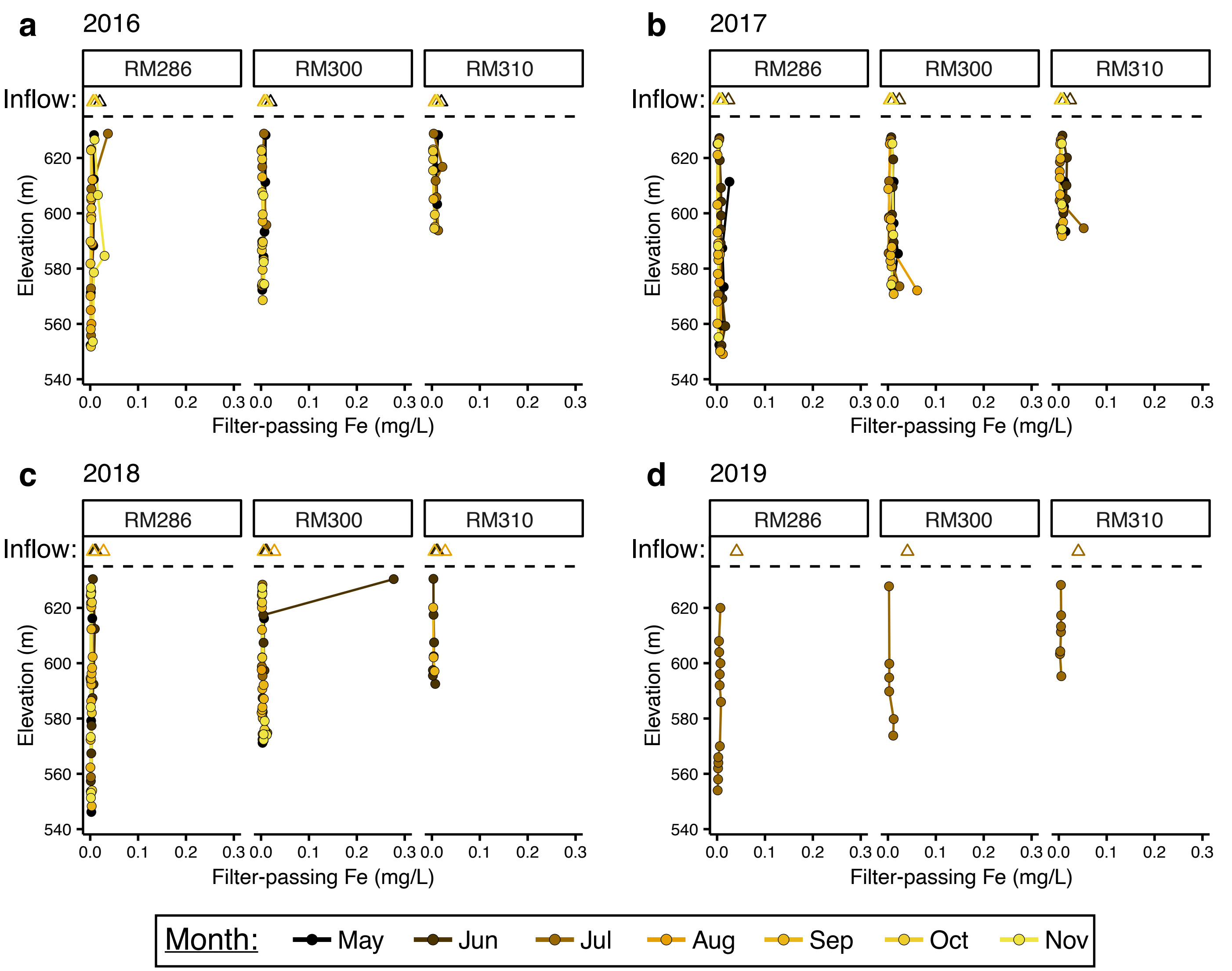

Figure S12. Filter-passing Fe concentrations at RM286, RM300, and RM310 in 2016 (**a**), 2017 (**b**), 2018 (**c**), and 2019 (**d**) in Brownlee Reservoir. Concentrations of filter-passing Fe in inflowing waters are represented by the open triangles above the profiles. Colors correspond to the month of collection. Some months included more than one sampling event, leading to multiple profiles with the same color. Lines connect points for samples from a single sampling event.

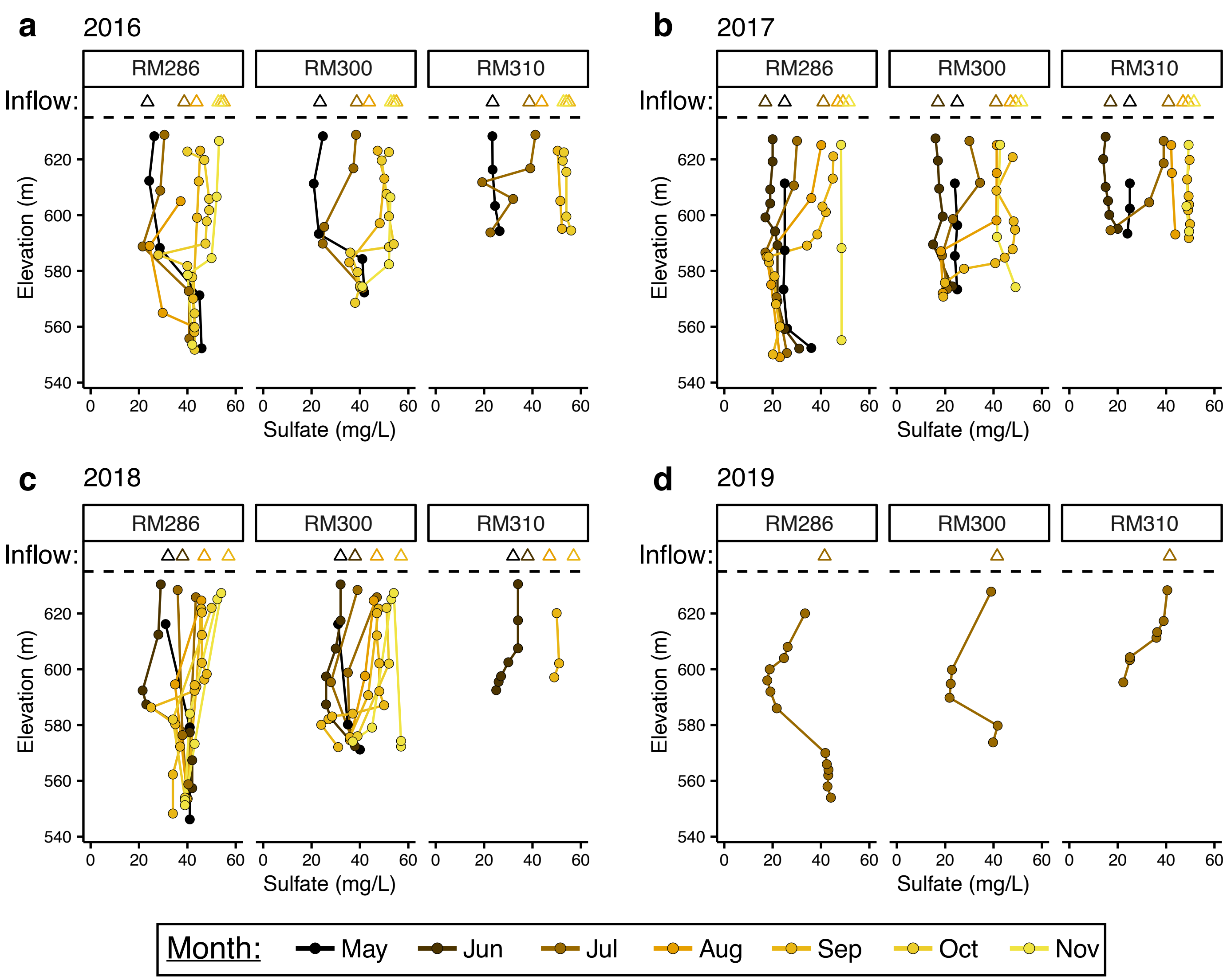

Figure S13. Sulfate concentrations in the Brownlee Reservoir water column at RM286, RM300, and RM310 in 2016 (**a**), 2017 (**b**), 2018 (**c**), and 2019 (**d**). Concentrations of sulfate in inflowing waters are represented by the open triangles above the profiles. Colors correspond to the month of collection. Some months included more than one sampling event, leading to multiple profiles with the same color. Lines connect points for samples from a single sampling event.

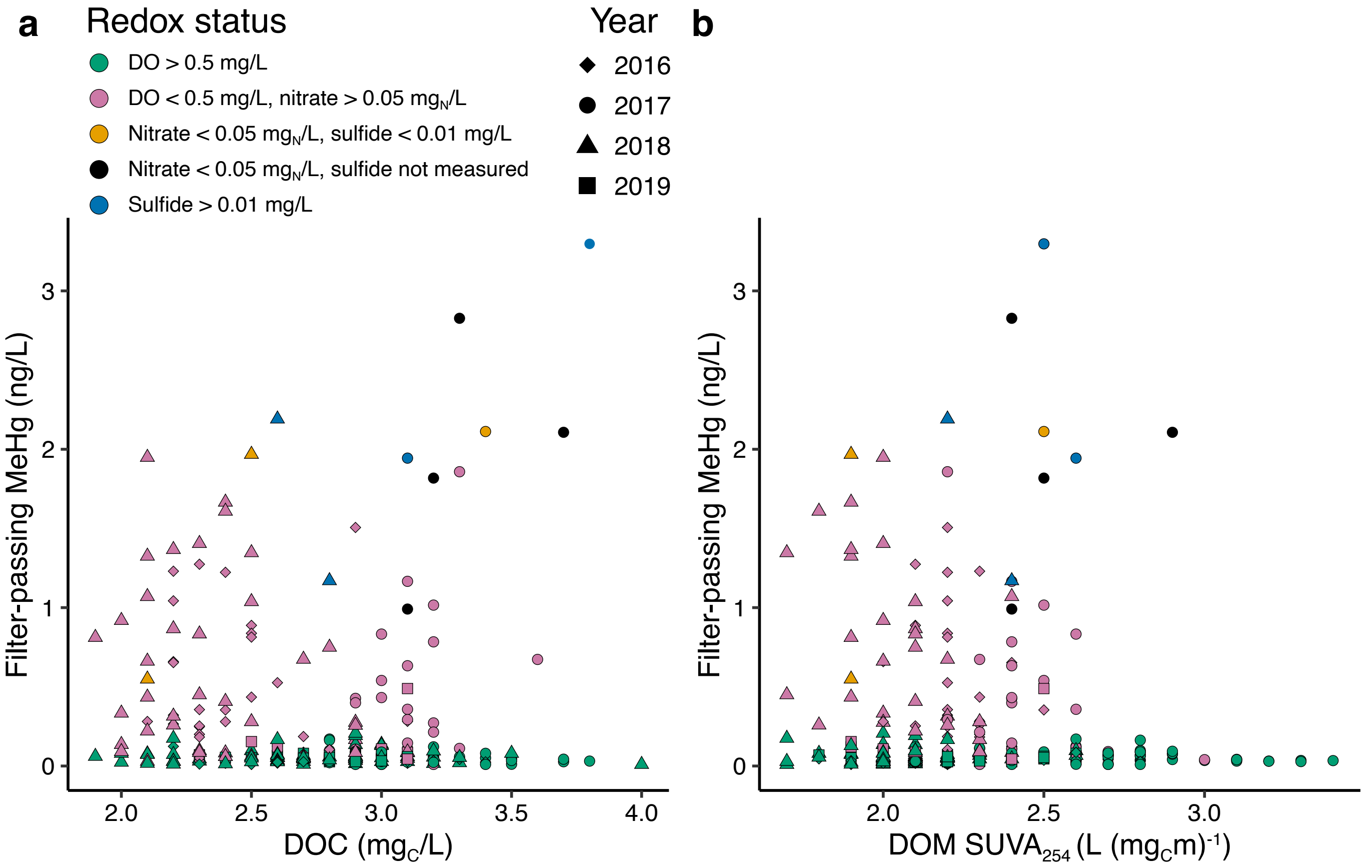

Figure S14. Filter-passing MeHg vs. dissolved organic carbon (DOC) concentration (**a**) or the specific UV absorbance at 254 nm of dissolved organic matter (DOM $SUVA_{254}$) (**b**). Color of the points corresponds to the redox status of the sample while the shape corresponds to the year it was collected.

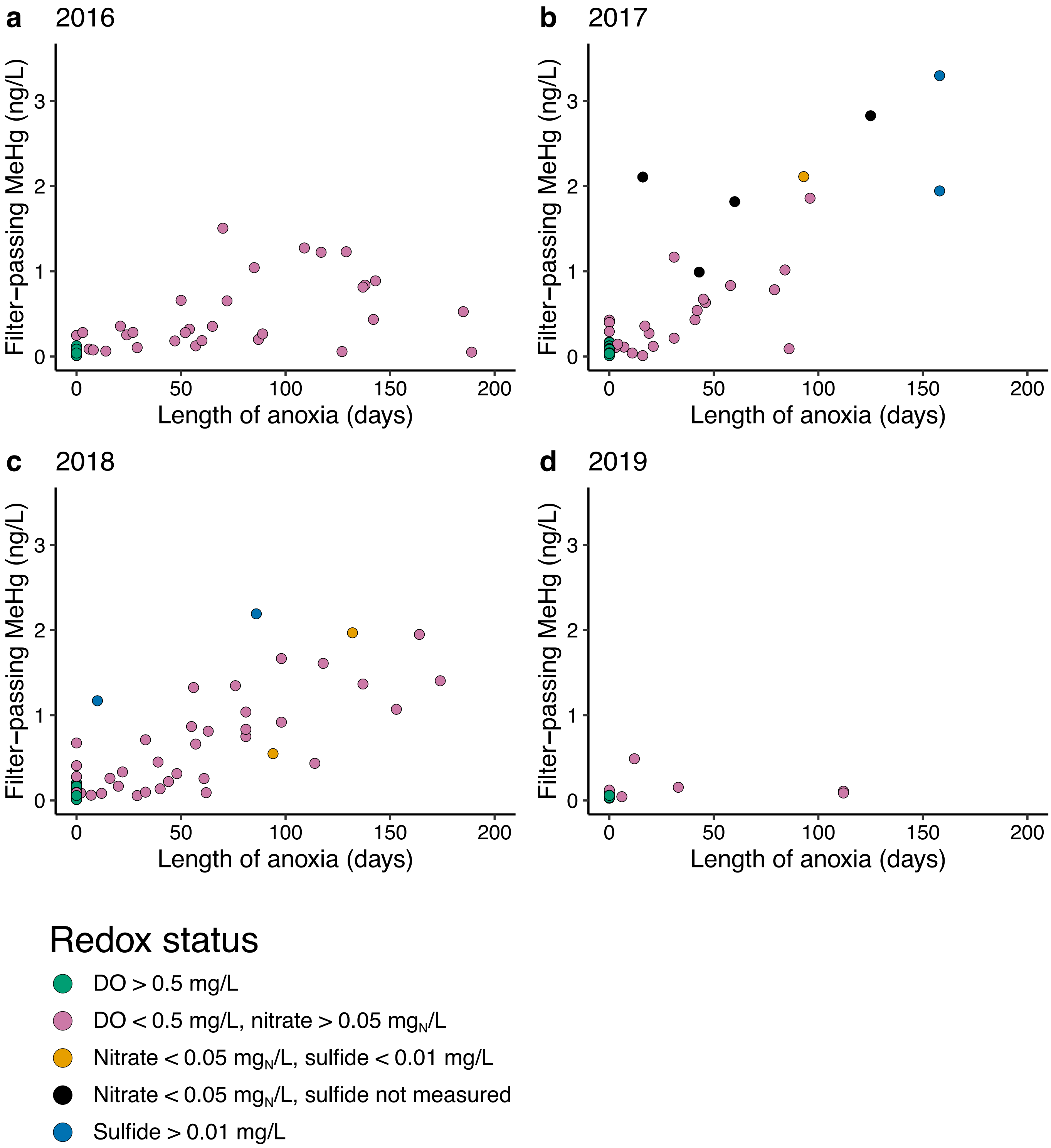

Figure S15. MeHg concentrations correlated to days of anoxia in a given water parcel in 2016 (**a**), 2017 (**b**), 2018 (**c**), and 2019 (**d**). Biweekly dissolved oxygen (DO) profiles were used to interpolate daily DO concentrations at each sampling location. Initial onset of anoxia at a given river mile and depth was determined as the day DO dropped below 0.5 mg/L.

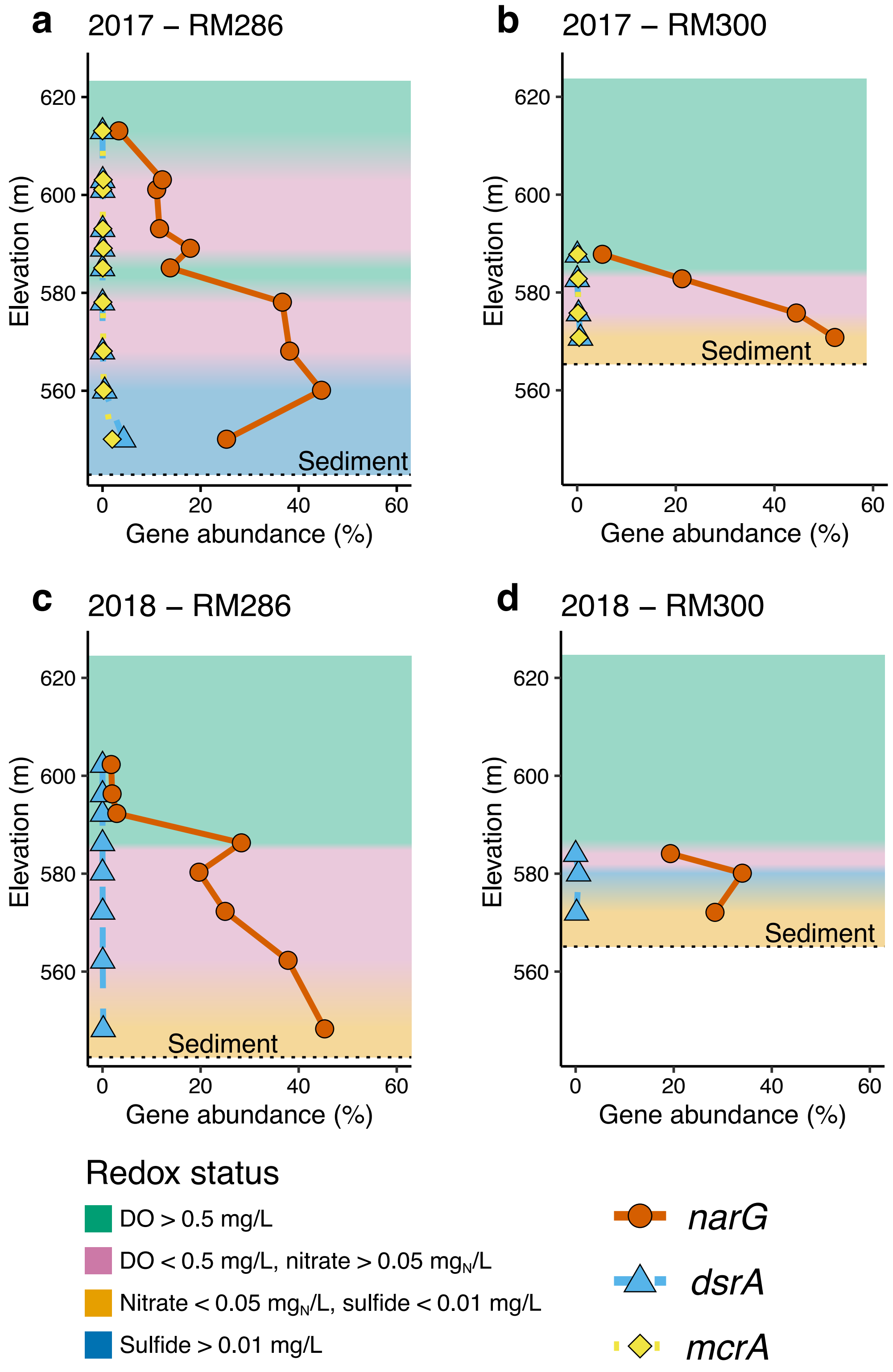

Figure S16. Depth profiles of representative genes for common terminal electron accepting processes (TEAPs) in 2017 (top panel, **a**,**b**) and 2018 (bottom panel, **c**,**d**) at RM286 (left side, **a**,**c**) and RM300 (right side, **b**,**d**). Gene abundance is presented at the read coverage of the gene relative to the median read coverage of 16 ribosomal protein genes. Background shading shows assigned redox status across the water column (Table 1). No *mcrA* genes were detected in 2018. Abbreviations: *narG* – dissimilatory nitrate reductase, representative of nitrate reduction; *dsrA* – reductive dissimilatory sulfite reductase, representative of sulfate reduction; *mcrA* – methyl coenzyme M reductase, representative of methanogenesis.

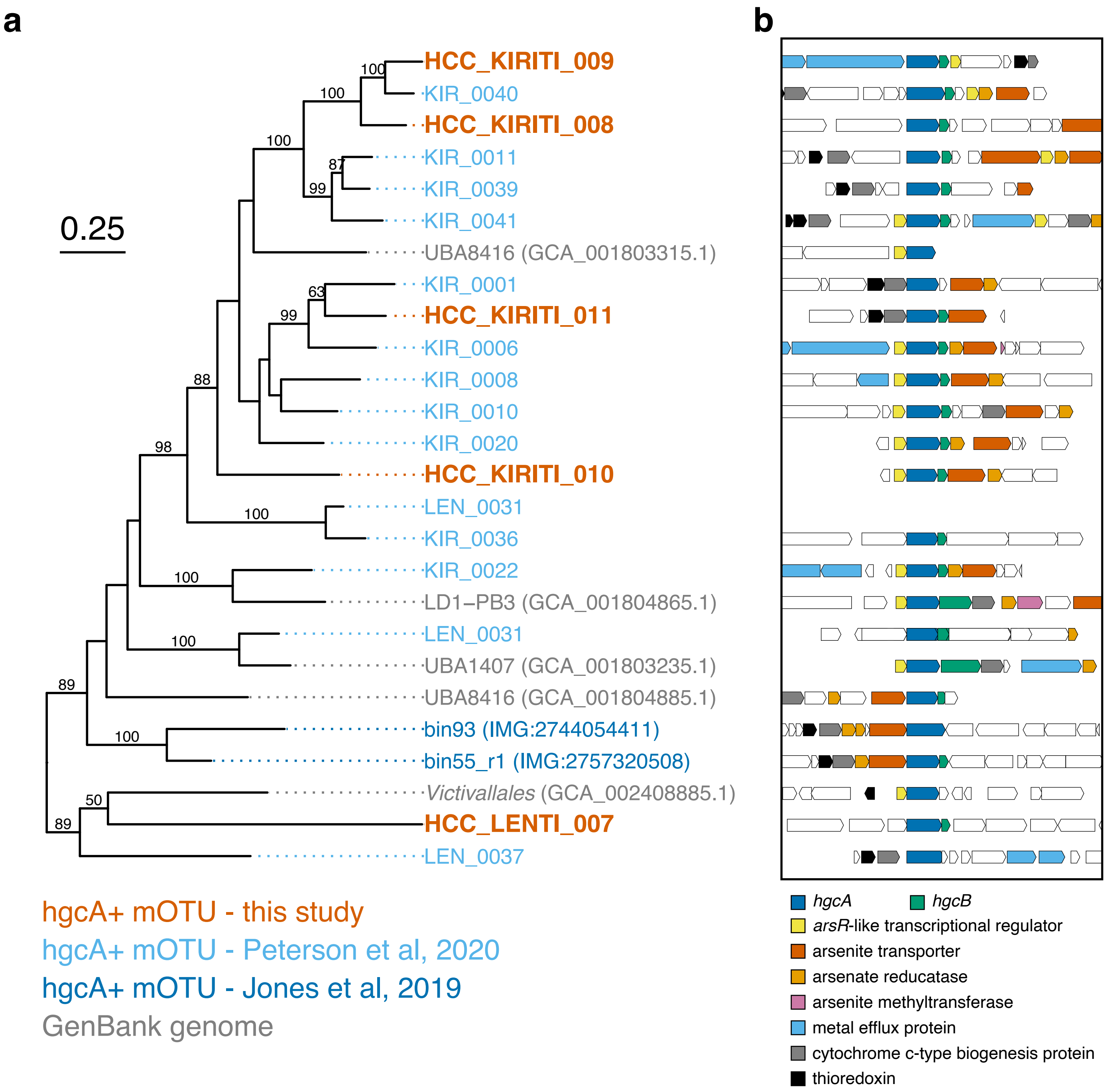

Figure S17: HgcA phylogeny (**a**) and gene neighborhoods (**b**) for *hgcA* genes within mOTUs associated with the *Kiritimatiellae* and *Lentisphaeria* families.

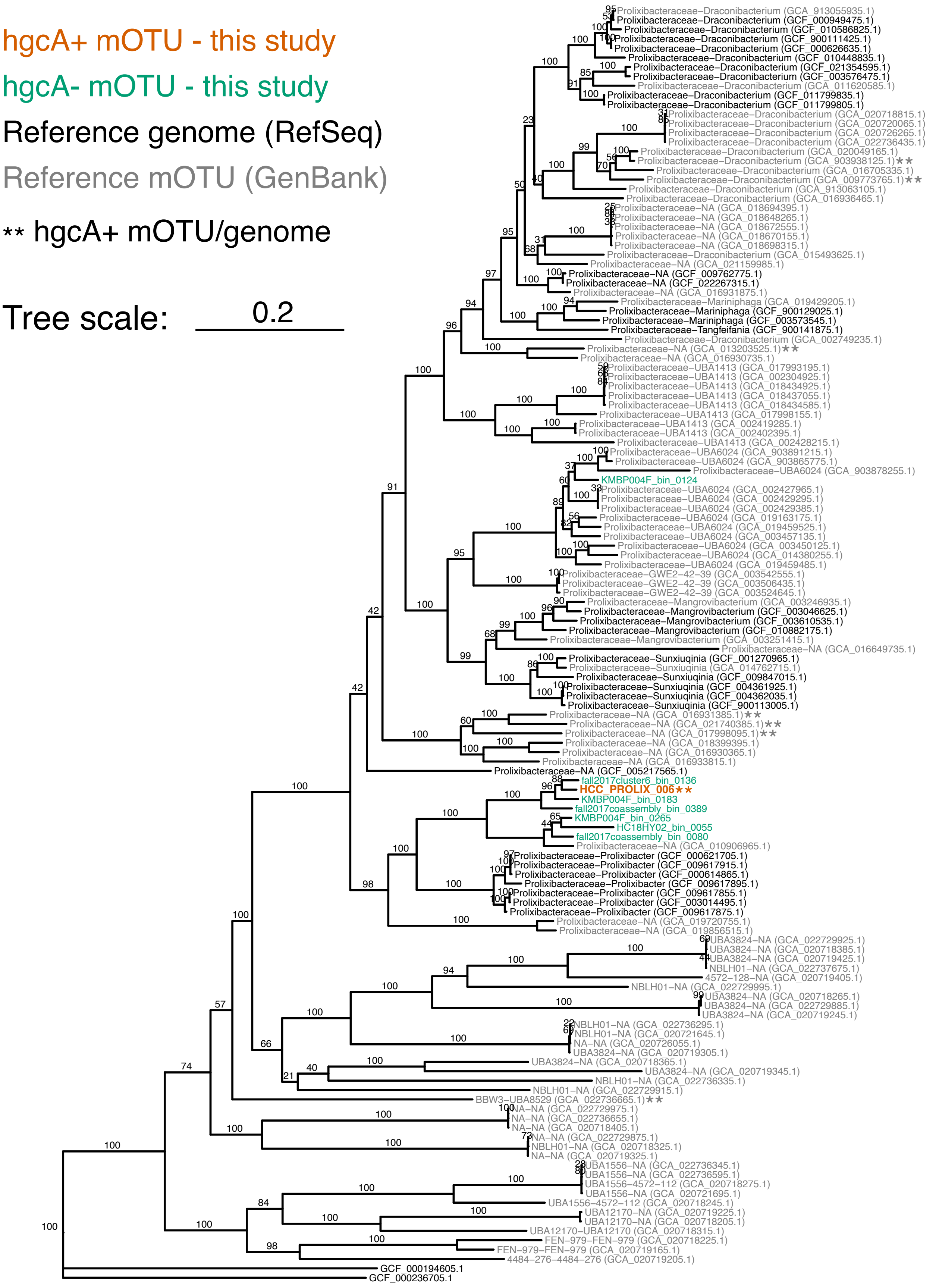

Figure S18. Phylogenetic tree of *Prolixibacteraceae* genomes. Tree is based on concatenated alignments of 16 ribosomal protein genes. Branch values represent branch support by rapid bootstrapping. All mOTUs and genomes in the NCBI GenBank and RefSeq databases that were annotated as *Prolixibacteraceae* (based on NCBI taxonomy) were included in this tree.

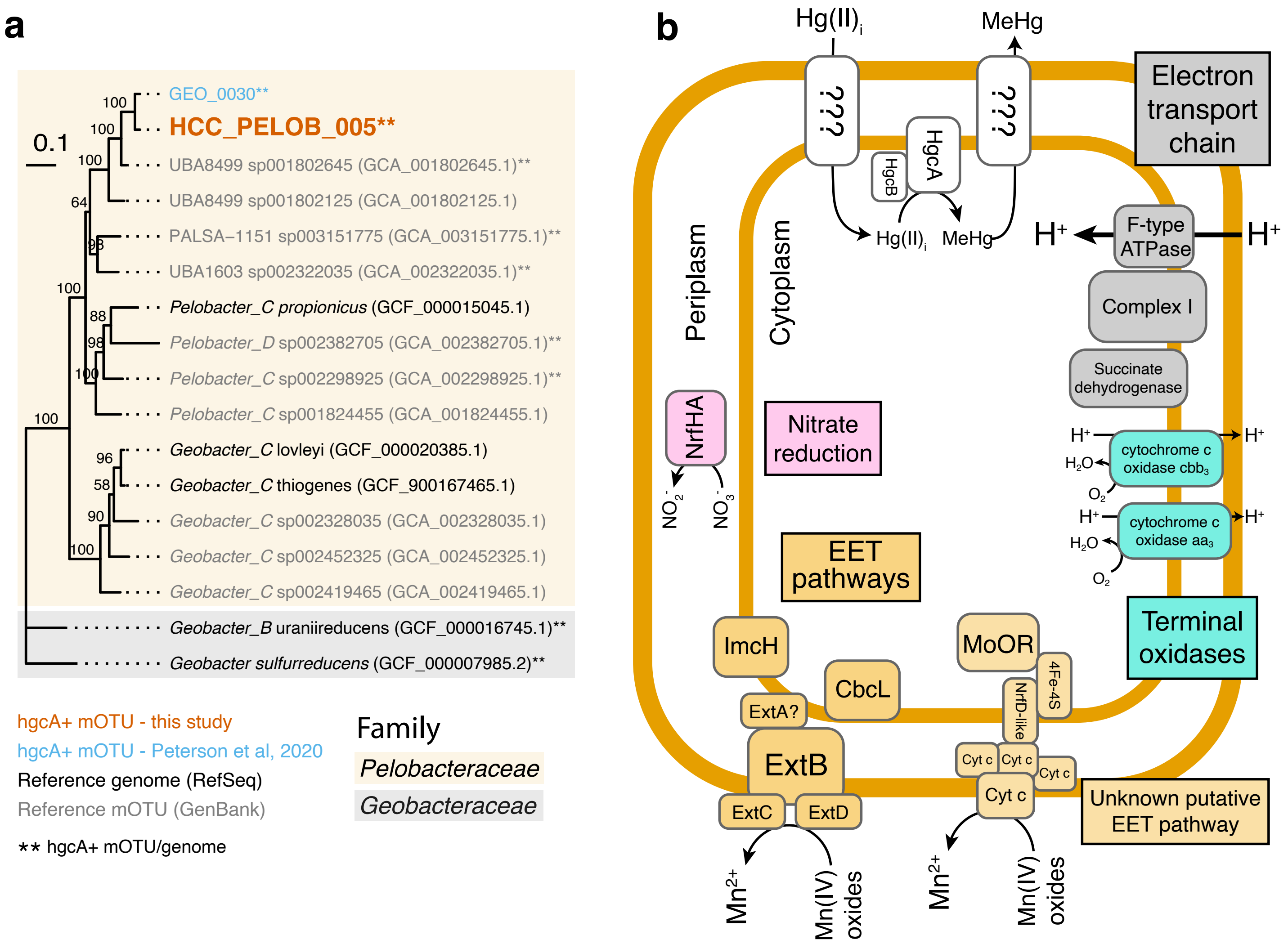

Figure S19. Phylogenetic (**a**) and metabolic (**b**) characterization of HCC_PELOB_005, an *hgcA*-containing mOTU in the *Pelobacteraceae* family. (**a**) Phylogenetic tree was constructed using genomes from RefSeq and GenBank databases and is based on 16 ribosomal proteins. One closely related genome (GEO_0030) from Peterson et al, 2020 is also included. (**b**) Cartoon diagram of terminal electron accepting pathways found in genome. “Cyt c” indicates the protein is homologous to an undescribed cytochrome c protein.

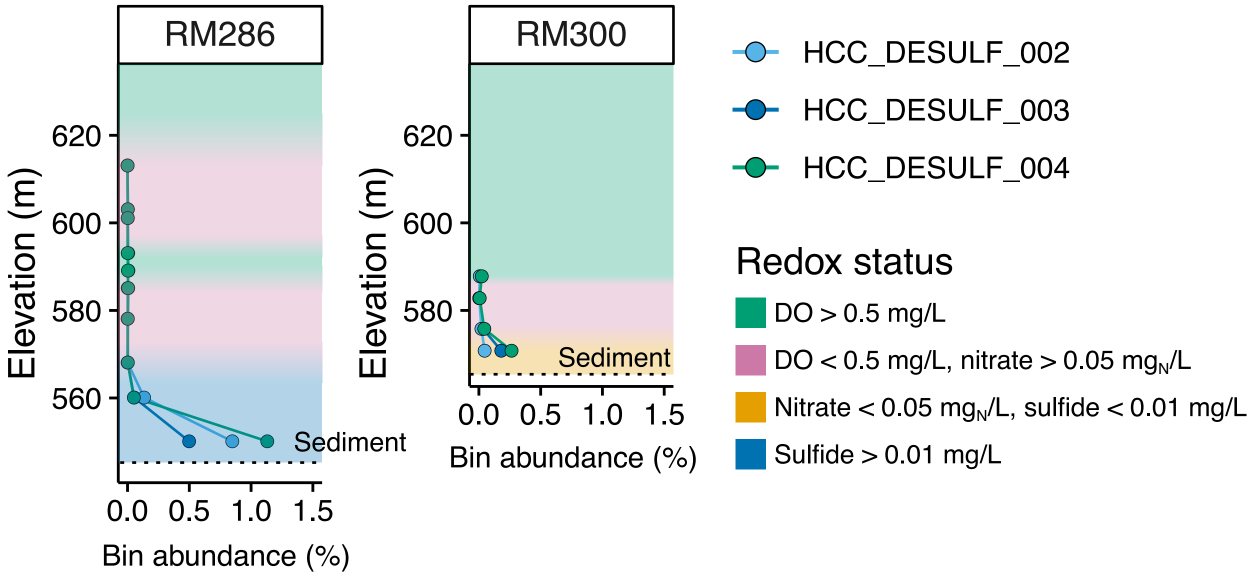

Figure S20: Abundance of hgcA+ mOTUs containing sulfate-reduction genes in 2017 metagenomes. Background shading represents the redox status assigned to the depths based on the water chemistry data.

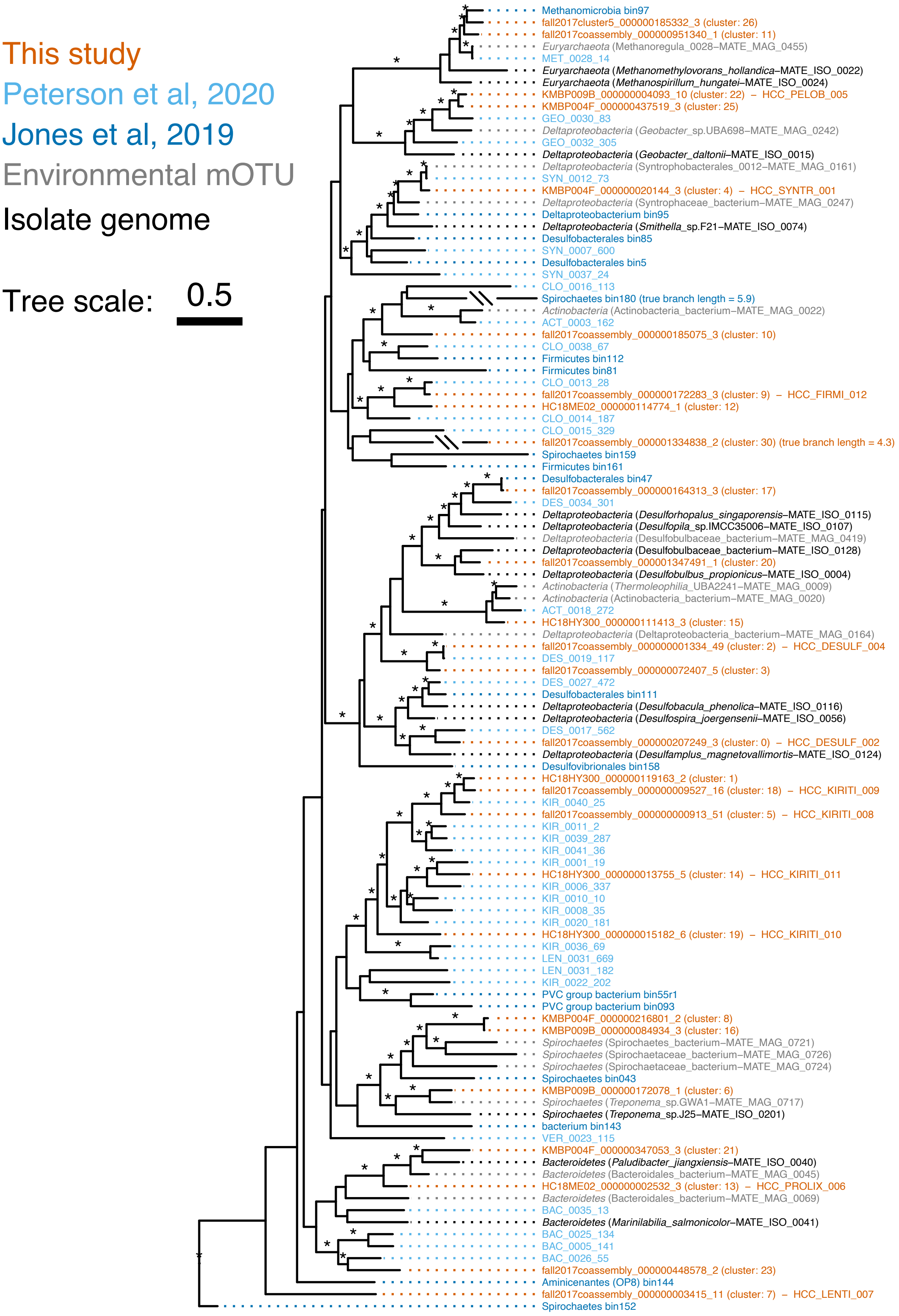

Figure S21: HgcA phylogeny for all *hgcA* genes identified in the assemblies. *hgcA* recovered in an mOTU include the mOTU name. All references were drawn from the Hg-MATE database. References from Hg-MATE that originated from Jones et al., 2019 or Peterson et al., 2020 are labeled with the original label from those studies. Colors indicate the source of the *hgcA* gene. Two terminal branches have been trimmed to improve readability and the true branch length added to the name. * indicates a bootstrap support value greater than 50.
